# Development and validation of a health and nutrition module for the project‐level Women's Empowerment in Agriculture Index (pro‐WEAI+HN)

**DOI:** 10.1111/mcn.13464

**Published:** 2022-12-08

**Authors:** Jessica Heckert, Elena M. Martinez, Greg Seymour, Audrey Pereira, Shalini Roy, Sunny S. Kim, Hazel Malapit, Gender Assets and Agriculture Project Phase 2 (GAAP2) Health and Nutrition Study Team

**Affiliations:** ^1^ Poverty, Health, and Nutrition Division International Food Policy Research Institute Washington District of Columbia USA; ^2^ CGIAR Research Program on Agriculture for Nutrition and Health International Food Policy Research Institute Washington District of Columbia USA; ^3^ Environment and Production Technology Division International Food Policy Research Institute Washington District of Columbia USA; ^4^ Present address: Friedman School of Nutrition Science and Policy Tufts University Boston Massachusetts USA; ^5^ Present address: Department of Public Policy University of North Carolina at Chapel Hill Chapel Hill North Carolina USA

**Keywords:** agency, gender equality, health indicators, nutrition indicators, nutrition‐sensitive programmes, women's empowerment

## Abstract

Agricultural development projects increasingly aim to improve health and nutrition outcomes, often by engaging women. Although evidence shows such projects can improve women's and children's health and nutrition and empower women, little is known about their impacts on women's health‐ and nutrition‐related agency and the extent to which impacts emerge through women's empowerment, largely due to a lack of instruments that measure the dimensions of women's agency that are directly relevant to health and nutrition outcomes. We developed an optional, complementary module for the project‐level women's empowerment in agriculture index (pro‐WEAI) to measure health‐ and nutrition‐related agency (pro‐WEAI + HN). Our method for developing related indicators used data collected from six agricultural development programmes implemented across Bangladesh, Burkina Faso and Mali (pooled sample = 12,114) and applied psychometric analysis (exploratory and confirmatory factor analysis) and the Alkire−Foster methodology. Results revealed seven indicators covering women's agency in the areas of her own health and diet; her health and diet during pregnancy; her child's diet; breastfeeding and weaning; purchasing food and health products; and acquiring food and health products. Multigroup confirmatory factor analysis revealed measurement invariance across contexts and samples. Tests of association (Cramer's V) and redundancy suggest that the pro‐WEAI + HN indicators measured aspects of agency that are distinct from the core pro‐WEAI. The uptake of these indicators in studies of nutrition‐sensitive agricultural development projects may strengthen the evidence on how such programming can enhance women's empowerment to improve health and nutrition outcomes for themselves and their children.

## Key messages

- We developed a health and nutrition module for the project‐level Women's Empowerment in Agriculture Index (pro‐WEAI + HN) to measure health‐ and nutrition‐related agency.
- We used data from six projects in Bangladesh, Burkina Faso and Mali; conducted psychometric analysis, and applied the Alkire−Foster methodology to validate seven standardized indicators.
- The seven pro‐WEAI + HN indicators measure aspects of agency that are conceptually and statistically distinct from the core pro‐WEAI indicators.
- Use of this module can strengthen the evidence on women's empowerment in the context of nutrition‐sensitive agriculture programmes and policies.

## 1 INTRODUCTION

The Sustainable Development Goals (SDGs) highlight the importance of improving gender equality and empowering women (SDG 5), ending hunger and malnutrition (SDG 2) and achieving good health for women and children (SDG 3). Agricultural development projects increasingly target these goals by incorporating gender‐sensitive and nutrition‐sensitive objectives to address the underlying determinants of malnutrition (Ruel & Alderman, 2013). Assessing the extent to which such projects can improve women's empowerment in health and nutrition requires appropriate indicators.

Recently, there have been significant advancements in the development of topic‐specific women's empowerment indices, such as the Women's Empowerment in Agriculture Index (WEAI) (Alkire et al., 2013), the project‐level WEAI (pro‐WEAI) (H. Malapit et al., 2019), the Women's Empowerment in Livestock Index (Galiè et al., 2018), the Survey‐based Women's Empowerment Index (Ewerling et al., 2017) and the Women's Empowerment in Nutrition Index (WENI) (Narayanan et al., 2019), along with the rigorous use of psychometric methods for validation (Cheong et al., 2017; Yount et al., 2019). However, there are no standardized measures of women's empowerment that focus specifically on nutritional outcomes, are validated in multiple contexts, and address lifecycle‐specific health and nutrition needs. Without standardized and topic‐specific measures, we cannot determine whether and how nutrition‐sensitive agriculture programmes contribute to women's empowerment and whether women's empowerment, in turn, leads to intended outcomes.

Numerous studies find evidence of cross‐sectional associations between women's empowerment and better diet and nutritional status among women (Amugsi et al., 2016; H. J. L. Malapit, Kadiyala et al., 2015; Malapit & Quisumbing, 2015; Sinharoy et al., 2018) and young children (van den Bold et al., 2013; Bose, 2011; Na et al., 2015; M. Shroff et al., 2009; M. R. Shroff et al., 2011). A growing body of evidence from impact assessments of nutrition‐ and gender‐sensitive agricultural development programmes finds that these programmes can both empower women and improve nutritional outcomes among women and children (Kumar et al., 2018; Olney et al., 2015, 2016), and that programme impacts on women's empowerment may lead to improved child nutritional outcomes (Heckert et al., 2019). However, a recent systematic review by Santoso et al. (2019) finds inconclusive relationships between women's empowerment and child nutrition, which they attribute to limitations in measurement and study design. Further research in this area depends on carefully operationalizing and measuring empowerment.

The agriculture‐nutrition pathways conceptual framework, which describes the multiple complex paths linking agriculture to nutrition, motivates our work and delineates processes that are proximate to nutritional outcomes, such as intrahousehold food allocation, from distal ones, such as access to credit or crop choice (Gillespie et al., 2012; Kadiyala et al., 2014). Distal factors are generally related to the productive sphere (production of goods and services that can be sold or are remunerated), whereas proximate ones are related to the reproductive or domestic sphere, which often goes unrecognized and uncompensated (e.g., child feeding, healthcare). All these pathways can be gendered, and women and men may have different degrees of power along them. Considering measures of and evidence for empowerment along these pathways, many of the more recently developed metrics focus on the productive sphere, and as we describe below, there is still a dearth of evidence on the relationships between empowerment in the domestic sphere and nutritional outcomes.

Existing evidence on the link between women's empowerment and nutrition outcomes draws on diverse metrics. Many studies focus on empowerment in the productive sphere, using WEAI (e.g., Gupta et al., 2019; H. J. L. Malapit, Kadiyala et al., 2015; Malapit & Quisumbing, 2015; Santoso et al., 2019). Other studies have analyzed the link between general empowerment in the domestic sphere and nutritional outcomes, using the Demographic and Health Surveys. For example, a general household decisions indicator was associated with lower stunting and wasting (India) (M. R. Shroff et al., 2011), increased use of healthcare services in a multicountry study (Ahmed et al., 2010), use of antenatal and postnatal care (India) (Mistry et al., 2009) and fully vaccinating children (Ethiopia) (Ebot, 2015). One study in Chad used a metric of mothers' input into child feeding decisions and found it was associated with a higher height‐for‐age *z*‐score (HAZ) (Bégin et al., 1999). Notably, there is a dearth of metrics on women's agency in the more proximate pathways in the agriculture‐nutrition conceptual framework.

One exception is the WENI, developed in India to measure women's agency around their own health and nutrition and includes agency items related to food, health and fertility (Narayanan et al., 2019). While it includes more domains for nutrition, it does not cover some key themes for nutrition‐sensitive agriculture, such as animal‐source foods, distribution of food within the home, acquiring key inputs, time use and child nutrition.

In this paper, we developed and validated the pro‐WEAI health and nutrition module (pro‐WEAI + HN), a survey module designed to measure women's instrumental agency in health and nutrition, and the indicators derived from the module. The module was designed to complement the core pro‐WEAI, which diagnoses areas of disempowerment, assesses project impact on women's empowerment in agricultural development projects and focuses primarily on productive work, especially agricultural production (H. Malapit et al., 2019). The health and nutrition indicators are intended to capture dimensions of empowerment that are distinct from—but complementary to—the core pro‐WEAI.

## 2 METHODS

### 2.1 Development of the questionnaire

Pro‐WEAI and pro‐WEAI + HN are being developed under the Gender, Agriculture, and Assets Project, Phase 2 (GAAP2), which brings together a portfolio of 13 agricultural development projects from nine countries. Participating projects helped develop the questionnaire, administered it in their impact evaluations and shared data to develop common indicators (H. Malapit et al., 2019).

Six nutrition‐sensitive agriculture projects in the GAAP2 portfolio elected to include pro‐WEAI + HN. At an inception workshop, which included local and international research and implementation teams, attendees collectively identified priority topics, indicators and survey items for pro‐WEAI + HN. It was determined that the module should have three specific characteristics. Firstly, it needed to address all three pillars of the food, health and care paradigm (United Nation's Children Fund, 1990), which projects applied to promote the consumption of nutritious foods, healthcare utilization and caregiving practices alongside agricultural production. Secondly, it needed to address key life stages—infancy, early childhood, pregnancy and lactation—when nutrition and health needs increase and when women's agency may be especially limited. Thirdly, it needed to consider animal‐source foods (eggs, milk and meat); several projects focused on homestead livestock production for meeting critical nutrient needs, and women often experience difficulty maintaining control over these high‐value resources. We do not include fruits and vegetables, because women do not experience the same limitations experienced with animal‐source foods (Kehoe et al., 2019). The module also drew inspiration from questions about women's decision‐making previously developed through qualitative field testing and used for the impact evaluation of an integrated maternal and child health programme in Haiti (Menon et al., 2002), which have been used in multiple impact evaluations (Kumar et al., 2018; Olney et al., 2016).

The module (Supporting Information: Appendix Table A1) was designed to be administered to women beneficiaries of nutrition‐sensitive agriculture programmes (or equivalent women in a baseline survey or control group). In the first section of the module on ‘Decisions’, respondents were asked about key health and nutrition decisions. For each of the 17 women's health and 13 child health decisions, respondents were asked about the normal decision‐makers for the activity (up to 3 individuals) and the extent to which they participated in the decision. The extent of input was asked because, in previous surveys that only asked who participates, most women reported participating, as women are often afforded some situational authority over domestic decisions. Moreover, it was not clear if participation meant they were acting on the request of others or were fully engaging in the decision. In the second section on ‘Products’, respondents were asked about obtaining 12 necessities (food, health products, clothing and toiletries). They were asked who generally makes the decision and whether they can usually acquire it when needed.

The results of cognitive interviews with 48 women in Bangladesh revealed that the questions were mostly well understood and provided insight into how to better word the questions; the full results of the study and the resulting questionnaire revisions are reported in Hannan et al. (2020). Supporting Information: Appendix Table A1 includes the revised wording. Owing to project timelines, five projects administered the survey using the original wording, and one used the revised version.

### 2.2 Application in projects and survey samples

The six GAAP2 projects that fielded the module were: (1) Agriculture, Nutrition, and Gender Linkages (ANGeL) in Bangladesh (Ahmed et al., 2017), (2) Food and Agricultural Approaches to Reducing Malnutrition (FAARM) in Bangladesh (Wendt et al., 2019), (3) Building Resilience of Vulnerable Communities, in Burkina Faso (Grameen) (Gash & Gray, 2016), (4) Soutenir l'Exploitation Famaliales pour Lancer l'Elevage des Volailles et Valoriser l'Economie Rurale (SELEVER) in Burkina Faso (Gelli et al., 2017), (5) Targeting and Realigning Agriculture to Improve Nutrition (TRAIN), in Bangladesh (Kumar and Ruel, 2020) and (6) Deploying Improved Vegetable Technologies to Overcome Malnutrition and Poverty, in Mali, (WorldVeg) (Schreinemachers et al., 2016). Supporting Information: Appendix Table A2 provides details on partners, focus, data collection, sampling strategies and ethical approvals.

The pooled sample was 12,114 (ANGeL = 3917, FAARM = 287, Grameen = 380, SELEVER = 1777, TRAIN = 5039, WorldVeg = 714). The sample was smaller for women who had been pregnant in the past 2 years or who had a child younger than 2 years old and when items were omitted from surveys. The combined sample includes women who are generally young (32% aged 16−24, 49% aged 25−34), have limited formal education (41% never attended school) and are married (98%) (Table 1). In the 2 years before the interview, 38% had been pregnant, and 32% had a child younger than 2 years old. Women who had never attended school were older, on average, compared to women who had attended school (results not shown), reflecting rapid educational expansion in these settings. The difference in these characteristics across the two regions largely reflects the sampling strategies. The Bangladesh studies sampled households with young children or where women were expected to become pregnant. In West Africa, Grameen and WorldVeg both sampled the general population of women, while SELEVER sampled households with young children. Each study obtained ethical approval from its respective institution(s).

**Table 1:** Characteristics of respondents

	(%)		
	All	Bangladesh	Burkina Faso and Mali
Age group			
16−24	32.0	41.9	17.1
25−34	48.5	52.4	42.6
35−44	13.3	4.3	26.8
45+	5.8	0.9	13.1
Missing	0.4	0.5	0.3
Education			
Never attended school	40.9	13.3	82.3
Less than primary	14.3	15.4	12.5
Primary	36.9	60.0	2.4
Secondary	7.1	11.3	0.7
Missing	0.8	0.0	2.1
Marital status			
Married	98.0	99.6	95.6
Unmarried (never married)	0.3	0.0	0.7
Unmarried (previously married)	1.6	0.4	3.3
Missing	0.2	0.0	0.4
Was pregnant or gave birth in last 2 years	38.0	35.1	40.8
Has child under age 2	31.6	19.2	40.0

*Note*: Weighted by the inverse of project sample size, so that characteristics are equally weighted by project sample. FAARM excluded question on whether woman has a child under age two. Age group, education and marital status data not available for Grameen. Missing data values for these categories only include cases from SELEVER and WorldVeg.

Abbreviation: FAARM, food and agricultural approaches to reducing malnutrition.

### 2.3 Data analysis

#### 2.3.1 Factor analyses to identify domains of indicators

To assess dimensionality, we conducted exploratory factor analysis (EFA) with a randomly selected half of the sample from the largest project in each region (TRAIN [Bangladesh] and SELEVER [Burkina Faso]). Respondents were eligible to respond to different items (i.e., all women, pregnant in the last 2 years, with a child less than 6 months, with a child less than 2 years; see Supporting Information: Appendix Table A1); thus, we conducted EFA separately for the ‘decisions’ and ‘products’ sections. For ‘decisions’, the sample was limited to women from dual‐adult households who had been pregnant in the last 2 years and had a child younger than 2 years. We used an ordinal variable of the extent of perceived participation in each joint decision; ‘not at all’ was coded as 1, ‘to a small extent’ as 2, ‘to a medium extent’ as 3 and ‘to a high extent’ as 4. Sole decision‐making was grouped with a high extent of participation.

Missing data were imputed using the expectation‐maximization algorithm (Graham, 2009), and EFA was conducted using the variance‐covariance matrices. For the items related to health and nutrition products (binary responses), the sample was limited to dual‐adult households, and EFA was based on tetrachoric correlation matrices. Scree plots and Eigenvalues were used to inform decisions about how many factors to retain. Both orthogonal (varimax) and oblique (oblimin) rotation options were considered to obtain a simple structure (each item loaded on a single factor). Items that loaded on multiple factors or <0.4 were dropped. EFA was conducted using Stata 15.0.

We used confirmatory factor analysis (CFA) to assess how well the factor structure suggested by the EFA fit the remaining TRAIN and SELEVER samples and the samples from the four other projects. When EFA suggested that a single factor in TRAIN was two factors in SELEVER, we conducted the CFA with two factors. The CFA samples were limited to respondents from dual‐adult households. Standardized coefficients were estimated, and full information maximum likelihood was used to include cases with missing responses. Model fit was assessed using the root mean square error of approximation (RMSEA), the comparative fit index (CFI) and the Tucker−Lewis index (TLI). Cut‐off values for the RMSEA (range 0−1) are <0.05 (good) and <0.08 (adequate). For the CFI and TLI (range 0−1), they are >0.95 (good) >0.90 (adequate). CFA was conducted using the lavaan and semTools packages in R 3.4.4.

To determine whether the constructs were similar across samples, we tested for measurement invariance between different project samples collected in the same country (Bangladesh and Burkina Faso) and region (West Africa) using multigroup CFA. We also assessed measurement invariance for mothers of children younger than 2 years and other women, for the domains that applied to both groups. For each comparison, five levels of measurement invariance were tested (L. Milfont & Fischer, 2010; Putnick & Bornstein, 2016):

(1) Configural invariance: same pattern of item loadings (factor structure) across groups.
(2) Weak, or metric, invariance: equal factor loadings across groups.
(3) Strong, or scalar, invariance: equal factor loadings and item intercepts across groups.
(4) Strict, or residual, invariance: equal factor loadings, item intercepts and residual variances (sum of item‐specific variance and error variance) across groups.
(5) Mean invariance: equal factor loadings, item intercepts, residual variance and latent factor means across groups.

Strong invariance (level 3) is considered sufficient for comparing the mean differences among latent constructs (Putnick & Bornstein, 2016) and adequate for our needs.

#### 2.3.2 Selection of indicators and cutoff values

We developed common indicators and cutoff values then using the Alkire−Foster methodology, which was used to develop multidimensional poverty indices and other WEAI indicators (Alkire & Foster, 2011). The indicators use the items for each dimension that emerged from the factor analysis. Additionally, our decisions were informed by the results of the cognitive interviewing of this module (Hannan et al., 2020). For example, many women reported that being asked the same questions regarding pregnancy and breastfeeding was unnecessarily repetitive, and the breastfeeding questions were dropped in this step.

To identify adequacy cutoffs for each indicator, we compared the proportion of women who would be identified as adequate at different cutoffs [i.e., any input (sole or joint to a small extent), medium input (sole or joint to a medium extent) or high input (sole or joint to a high extent), into the decisions for each factor; decides to purchase or has access to 25%, 50%, 75% or 100% of products]. Decisions regarding the cutoff values were informed by identifying large shifts in the percentage classified as adequate for alternative values and by normative reasoning (consistent with theory and known information) as suggested by Alkire et al. (2015). We also examined the associations between *decision to purchase* and *has access to* each item to determine if there was justification for combining them.

To assess the prevalence of empowerment, we calculated the mean adequacy value of each indicator, pooled by region. To compare means according to women's age group and education level, we used one‐way analysis of variance (ANOVA) and post‐ANOVA contrasts to identify significant differences between adjacent ordinal categories. Alkire et al. (2015) typically recommend assessing the intensity of empowerment (i.e., the average proportion of indicators on which an empowered individual is empowered) and dimensional monotonicity (which would require that empowerment increase if an empowered person who is not yet empowered in all dimensions becomes empowered in an additional dimension). However, because some indicators are specific to subpopulations (e.g., pregnant women), we do not aggregate the seven indicators that we derive and thus cannot assess the intensity or dimensional monotonicity.

#### 2.3.3 Tests of association between indicators

To assess the strength of association among the new indicators and between the seven new indicators and 12 core pro‐WEAI indicators, we conducted pairwise comparisons using Cramer's V, calculated as a percentage of the maximum possible variation (Alkire et al., 2015). We also assessed redundancy between each pair of indicators (percentage of respondents inadequate on both indicators). For two indicators, A and B, redundancy is the number of people inadequate in both, divided by the number of people inadequate in A, where A is the indicator in which fewer people are inadequate (Alkire et al., 2015). We rely on estimates of association and redundancy to assess discriminant validity, as the structure of the data (i.e., skip patterns and construction of a binary indicator) does not permit formal testing (Furr, 2018).

#### 2.3.4 Ethics statement

This study was based on secondary data analysis. The six projects that shared data for analysis all received ethical approval from the institutional review boards of their respective institutions.

## 3 RESULTS

We examined results for each project separately but reported results by region. In pooled means, values are weighted by the inverse of the project sample size, so that projects are equally weighted. The means of the items used in the EFAs and CFAs are reported in Supporting Information: Appendix Tables A3 and A4, while the respective correlation matrices are reported in Supporting Information: Appendix Tables A5, A6, A10 and A11.

### 3.1 Domains of health and nutrition agency: EFA

EFA results from the ‘decisions’ section using TRAIN data suggested a three‐factor solution: *decides on own health and diet during pregnancy and lactation*, *decides on child's health and diet* and *decides to seek healthcare* (Supporting Information: Appendix Table A7). Results using data from SELEVER suggested a four‐factor solution, the *own health and diet* and *healthcare* factors were similar to TRAIN. The items related to child health and diet, however, loaded on separate factors in SELEVER: one on feeding children animal‐source foods and the other about weaning and breastfeeding decisions. One explanation is that in this part of West Africa, where extended periods of postpartum abstinence are often tied to breastfeeding (Bongaarts et al., 1984), decisions about breastfeeding and weaning may be more strongly related to sex and fertility decisions than child‐feeding concerns.

Additionally, some items on women's health and diet were administered to all women, whereas others targeted recently pregnant women. So that the indicators could be used in a broader range of samples, we decided to split this dimension into two different factors—one for decisions unrelated to pregnancy and lactation and one focused on decisions during pregnancy and lactation. Additionally, we dropped the ‘during breastfeeding’ questions. This decision was motivated by the cognitive interviewing results that women found the ‘during pregnancy’ and ‘during breastfeeding’ questions repetitive (Hannan et al., 2020), the high correlation between the individual pregnancy and breastfeeding questions (Supporting Information: Appendix Table A5), the fact that pregnancy is a more salient marker for aiding recall (Bradburn et al., 1987), and because not all women breastfeed.

EFA results for the ‘products’ items suggested a two‐factor solution for both TRAIN and SELEVER. One factor described *decides about purchasing health and nutrition products*; the other described *has access to health and nutrition products* (Supporting Information: Appendix Table A8). To further consider the need for two factors, we examined the correspondence between the two items at the product level; for most products, around one‐fourth of women had access to each product, but could not decide to purchase it (Supporting Information: Appendix Table A9).

These results led us to conduct the CFA which we separately tested, due to the slightly different samples, a five‐factor model related to health and nutrition decisions: *decides on own health and diet*, *decides on health and diet during pregnancy*, *decides on child's diet*, *decides on weaning and breastfeeding*, *decides to seek healthcare* and two‐factor model related to health and nutrition products: *decides to purchase food and health products* and *has access to food and health products*.

### 3.2 Identifying domains: CFA

The CFA results from testing the five‐factor structure for ‘decisions’ items using CFA, led us to drop three items that did not consistently load: ‘feeding a sick child’, ‘having another child’ and ‘using contraception.’ A few additional items loaded poorly for the Grameen data set but were not dropped, because the sample was smaller, and many respondents did not have young children. After dropping these items, the five‐factor structure for the ‘decisions’ items fit well for all five projects (Table 2). (FAARM data were not included in this step, because too many items were omitted from the survey).

**Table 2:** Standardized factor loadings from CFA of woman and child health and nutrition items

		Standardized factor loading				
Latent factor	Decision	TRAIN	ANGeL	SELEVER	Grameen	WorldVeg
Decides on own health and diet	Resting when ill	0.77	0.54	0.71	*	0.92
	Foods to prepare	0.70	0.49	0.56	0.92	0.75
	Foods to eat	0.65	0.48	0.65	0.92	0.77
Decides on own health and diet during pregnancy	Work during pregnancy	0.79	0.71	0.66	*	0.79
	Rest during pregnancy	0.82	0.69	0.65	*	0.82
	Eggs during pregnancy	0.87	0.95	0.92	0.94	0.95
	Milk during pregnancy	0.88	0.98	0.90	0.93	0.96
	Meat/poultry/fish during pregnancy	0.82	0.96	0.87	0.84	0.95
Decides on child's diet	Feeding child eggs	0.86	0.86	0.88	0.79	0.87
	Feeding child milk	0.85	0.89	0.97	0.88	0.86
	Feeding child meat/poultry/fish	0.83	0.89	0.95	0.95	0.89
	Feeding sick child	0.79	0.72	*	*	0.79
Decides on weaning and breastfeeding	Breastfeeding child	0.63	0.89	0.93	0.72	0.92
	Ending breastfeeding	0.65	0.53	0.94	*	0.93
	Complementary foods	0.81	0.65	0.84	0.35	0.61
Decides to seek healthcare	Doctor when ill	0.75	0.74	0.79	0.77	0.83
	Having another child	0.68	0.66	*	*	*
	Using contraception	0.66	0.65	*	*	*
	Doctor when pregnant	0.81	0.64	0.78	0.78	0.76
	Sick child to doctor	0.85	0.74	0.77	0.89	0.78
	Child well visits	0.80	0.77	0.75	0.89	0.75
	Total observations	2382	3715	867	380	663
	Observations used	2382	3715	867	378	661
	CFI	0.961	0.950	0.951	0.948	0.94
	TLI	0.950	0.936	0.937	0.934	0.924
	RMSEA	0.046	0.061	0.062	0.060	0.081

*Note*: CFA models include respondents from dual‐adult households only and were run using standardized estimates and full information maximum likelihood estimation. Data from FAARM were not included, because a large number of items were not included in the survey. Each item was only allowed to load on a single factor.

Abbreviations: CFA, confirmatory factor analysis; CFI, comparative fit index; FAARM, food and agricultural approaches to reducing malnutrition; RMSEA, root mean square error of approximation; TLI, Tucker−Lewis index.

*Item dropped from the CFA because it did not load.

In testing a two‐factor structure for the ‘products’ items, clothing for children and self were omitted due to low factor loadings. The revised CFA models fit well for all six project samples (Table 3).

**Table 3:** Standardized factor loadings from CFA of health products items

Latent factor	Health product	TRAIN	FAARM	SELEVER	Grameen	WorldVeg	ANGeL
Decides to purchase food and health products	Small foods	0.81	0.64	0.69	0.65	0.91	0.79
	Large foods	0.84	0.51	0.68	0.62	0.84	0.78
	Eggs	0.88	0.87	0.86	0.76	0.88	0.91
	Milk	0.89	–	0.85	0.59	0.87	0.95
	Meat/poultry/fish	0.89	0.67	0.83	0.75	0.88	0.91
	Medicine for child	0.62	0.70	0.72	0.55	0.89	0.57
	Medicine for self	0.61	0.69	–	0.49	0.90	0.60
	Toiletries	0.67	0.77	0.55	0.29	0.88	0.62
Has access to food and health products	Small foods	0.85	0.64	0.59	0.42	0.51	0.70
	Large foods	0.78	0.44	0.56	0.14	0.72	0.60
	Eggs	0.84	0.70	0.72	0.54	0.70	0.59
	Milk	0.87	–	0.70	0.72	0.81	0.69
	Meat/poultry/fish	0.86	0.68	0.74	0.77	0.83	0.63
	Medicine for child	0.89	0.73	0.58	0.15	0.78	0.84
	Medicine for self	0.85	0.68	–	0.24	0.76	0.90
	Toiletries	0.83	0.75	0.44	0.27	0.56	0.75
	Total observations	2383	287	867	380	681	3715
	Observations used	2383	287	866	380	680	3715
	CFI	0.991	0.908	0.956	0.931	0.989	0.987
	TLI	0.988	0.872	0.941	0.902	0.984	0.979
	RMSEA	0.038	0.100	0.060	0.078	0.045	0.048

*Note*: CFA models include respondents from dual‐adult households only and were run using standardized estimates and full information maximum likelihood estimation. Each item was only allowed to load on a single factor.

Abbreviations: CFA, confirmatory factor analysis; CFI, comparative fit index; RMSEA, root mean square error of approximation; TLI, Tucker−Lewis index.

^–^Item omitted by project.

### 3.3 Results of measurement invariance tests

Results from the multigroup CFA, which compared the measurement structure across projects, showed strong measurement invariance (level 3) when comparing the five‐factor latent model for ‘decisions’ for the two projects in Burkina Faso, the projects in Burkina Faso and Mali, and two projects in Bangladesh (Table 4). For the ‘products’ section, the results met the qualifications for mean measurement invariance (level 5) for the three projects in Bangladesh and strong measurement invariance (level 3) for the two projects in Burkina Faso and the three projects in West Africa (Burkina Faso and Mali).

**Table 4:** Tests of measurement invariance between projects in the same country/region for health products factors and women and children health and nutrition decisions factors

	Health product factors			Women and child health and nutrition decisions factors		
Test statistic	Bangladesh (ANGeL, FAARM and TRAIN)	Burkina Faso (Grameen and SELEVER)	Burkina Faso/Mali (Grameen, SELEVER and WorldVeg)	Bangladesh (ANGeL and TRAIN)	Burkina Faso (Grameen and SELEVER)	Burkina Faso/Mali (Grameen, SELEVER and WorldVeg)
Overall						
CFI	0.988	0.940	0.975	0.961	0.940	0.931
TLI	0.977	0.916	0.958	0.947	0.922	0.910
RMSEA	0.051	0.078	0.060	0.055	0.070	0.077
Configural invariance						
CFI	0.988	0.936	0.975	0.949	0.910	0.925
RMSEA	0.051	0.086	0.063	0.081	0.106	0.100
Weak invariance						
CFI	0.983	0.932	0.964	0.939	0.906	0.910
RMSEA	0.057	0.085	0.071	0.086	0.105	0.106
Strong invariance						
CFI	0.980	0.913	0.933	0.925	0.904	0.900
RMSEA	0.058	0.091	0.092	0.093	0.103	0.108
Strict invariance						
CFI	0.963	0.859	0.855	0.885	0.833	0.859
RMSEA	0.074	0.110	0.126	0.112	0.131	0.123
Mean invariance						
CFI	0.960	0.831	0.829	0.881	0.830	0.857
RMSEA	0.076	0.120	0.136	0.113	0.131	0.122

*Note*: Multigroup CFA models include respondents from dual‐adult households only and were run using standardized estimates and full information maximum likelihood estimation.

Abbreviations: CFA, confirmatory factor analysis; CFI, comparative fit index; RMSEA, root mean square error of approximation; TLI, Tucker−Lewis index

In the multigroup CFA results comparing mothers and non‐mothers (of young children), there was evidence of mean measurement invariance (level 5) for the health product items in the ANGeL, SELEVER, TRAIN and WorldVeg datasets (Supporting Information: Appendix Table A12). Results did not demonstrate invariance between mothers and non‐mothers in the FAARM and Grameen projects, but as both samples are relatively small, it is difficult to draw conclusions.

### 3.4 Establishing cutoffs

A comparison of potential cutoff criteria showed large shifts in the percentage classified as adequate when comparing ‘medium’ and ‘high’ input (Supporting Information: Appendix Figure A1). Additionally, a ‘high input’ threshold could discount joint decision‐making. Thus, for each of the five indicators on health and nutrition decision‐making, women were considered adequate if, for all activities related to that indicator, they made decisions solely, they participated in joint decisions to at least a medium extent or the decision was not applicable (Table 5).

**Table 5:** Pro‐WEAI health and nutrition module indicators

Indicator	Decisions/inputs used	Adequate if respondent…
Decides on own health and diet	Input into decisions to: Rest when ill; Foods to prepare; Foods to eat.	Is the sole decision‐maker, contributes jointly at least ‘to a medium extent’, or decision is not applicable for all decisions
Decides on health and diet during pregnancy	Input into decisions to…during pregnancy: Work; Rest; Eat eggs; Consume milk or milk products; Eat meat.	Is the sole decision‐maker, contributes jointly at least ‘to a medium extent’, or decision is not applicable for all decisions
		(Only for women who have been pregnant or given birth in the past 2 years)
Decides on child's diet	Input into decisions to: Feed child eggs; Feed child milk and milk products; Feed child meat.	Is the sole decision‐maker, contributes jointly at least ‘to a medium extent’, or decision is not applicable for all decisions
		(Only for women with children <2 years old)
Decides on weaning and breastfeeding	Input into decisions: Whether to breastfeed; When to wean; To start giving other foods.	Is the sole decision‐maker, contributes jointly at least ‘to a medium extent’, or decision is not applicable for all decisions
		(Only for women with children <2 years old)
Decides to seek healthcare	Input into decisions to: Go to the doctor when ill; Go to the doctor when pregnant; Take sick child to the doctor; whether to take child for well visits.	Is the sole decision‐maker, contributes jointly at least ‘to a medium extent’, or decision is not applicable for all decisions
Decides to purchase food and health products	Input into purchasing: Small quantities of food; large quantities of food; eggs; milk (or milk products); meat, poultry, or fish; medicines for child; medicines for self; toiletries.	Contributes to decision (sole or joint) or response is not applicable to all items
Has access to food and health products	Able to acquire: Small quantities of food; large quantities of food; eggs; milk (or milk products); meat, poultry, or fish; medicines for child; medicines for self; toiletries.	Responds ‘Yes’ or response is not applicable to all items

Abbreviation: Pro‐WEAI, project‐level Women's Empowerment in Agriculture Index.

For the two health product indicators, a comparison of thresholds revealed that the strictest of 100% was achieved by half of the women for *decides to purchase food and health products*, and two‐thirds for *has access to food and health products* (Supporting Information: Appendix Figure A1). The selection of all products as the cutoff value was determined because all products are essential needs, and the cutoff leaves room for improvement. Thus, women were considered adequate in *decides to purchase food and health products* if they participated in decisions, either solely or jointly, about all products, except for those not applicable (Table 5). Similarly, women were adequate in *has access to food and health products* if they could access all products if needed, except for those not applicable.

**Figure 1:**
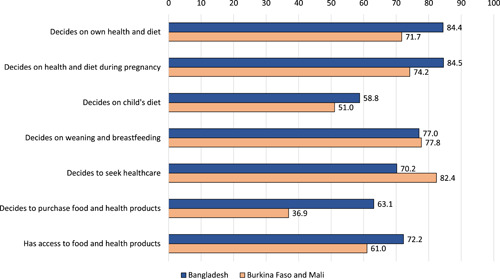
Percent of women achieving adequacy, by indicator and region. *Source*: Project data from Bangladesh: ANGeL (*N* = 3917), FAARM (*N* = 287) and TRAIN (*N* = 5040); and Burkina Faso and Mali: Grameen (*N* = 380), SELEVER (1777) and WorldVeg (*N* = 714). Weighted by inverse project sample size. Each of the seven indicators presented is comprised of at least three items.

Figure 1 reports the percentage of women achieving adequacy on each indicator by region. The mean differences may be attributable to the different sampling strategies, rather than regional differences. The Bangladesh studies identified women who were mothers of young children or likely to become pregnant; the Burkina Faso and Mali projects sampled a broader range of women. Adequacy on *decides on own health and diet* and *decides on own health and diet during pregnancy* were 72% and 74%, respectively in Burkina Faso and Mali and 84% and 85%, respectively, in Bangladesh. A little over half of the women in each region achieved adequacy on *decides on child's diet* in both regions, and a little over three‐quarters were adequate on *decides on weaning and breastfeed*. The adequacy levels were 70% (Bangladesh) and 82% (Burkina Faso and Mali) for *decides to seek healthcare*; 63% and 37% for *decides to purchase food and health products*; and 72% and 61% for *has access to food and health products*.

### 3.5 Associations among health and nutrition indicators

The associations (Cramer's V) between each pair of indicators reveal that the highest are among the five ‘decisions’ (Table 6). Associations between these five and the ‘product’ indicators are generally lower (V < 0.25), particularly in the Burkina Faso and Mali projects. Across all projects, associations are higher (V < 0.50) for *decides on own health*, *decides on own health and diet during pregnancy* and *decides on weaning and breastfeeding*. In Burkina Faso and Mali, *decides on health and diet during pregnancy* is strongly associated with *decides on child's diet*, although this is not the case in Bangladesh. This may be because mothers‐in‐law may have considerable influence on child diet in Bangladesh.

**Table 6:** Associations (Cramer's V) among health and nutrition indicators

		Decides on own health and diet	Decides on health and diet during pregnancy	Decides on child's diet	Decides on weaning and breastfeeding	Decides to seek healthcare	Decides to purchase food and health products	Has access to food and health products
All projects	Decides on own health and diet	1.00						
	Decides on health and diet during pregnancy	0.50	1.00					
	Decides on child's diet	0.16	0.40	1.00				
	Decides on weaning and breastfeeding	0.35	0.57	0.39	1.00			
	Decides to seek healthcare	0.38	0.49	0.10	0.29	1.00		
	Decides to purchase food and health products	0.20	0.17	0.06	0.11	0.08	1.00	
	Access to food and health products	0.16	0.16	0.12	0.12	0.05	0.29	1.00
Bangladesh	Decides on own health and diet	1.00						
	Decides on health and diet during pregnancy	0.47	1.00					
	Decides on child's diet	0.18	0.34	1.00				
	Decides on weaning and breastfeeding	0.35	0.50	0.29	1.00			
	Decides to seek healthcare	0.46	0.50	0.04	0.32	1.00		
	Decides to purchase food and health products	0.24	0.20	0.14	0.12	0.18	1.00	
	Access to food and health products	0.20	0.17	0.21	0.15	0.11	0.34	1.00
Burkina Faso and Mali	Decides on own health and diet	1.00						
	Decides on health and diet during pregnancy	0.56	1.00					
	Decides on child's diet	0.24	0.68	1.00				
	Decides on weaning and breastfeeding	0.33	0.75	0.61	1.00			
	Decides to seek healthcare	0.41	0.50	0.15	0.25	1.00		
	Decides to purchase food and health products	0.05	0.12	0.06	0.12	0.10	1.00	
	Access to food and health products	0.08	0.09	0.04	0.04	0.04	0.17	1.00

*Note*: Each of the seven indicators presented is comprised of at least three items.

The highest redundancy is between *decides to seek healthcare* and both *decides on own health and diet* and *decides on own health and diet during pregnancy*, indicating that women who are disempowered in decisions on their own health and diet, including during pregnancy, are also likely to be disempowered in the freedom to seek healthcare (Supporting Information: Appendix Table A13). In contrast, there is low redundancy between *has access to food and health products* and the following three: *decides on own health and diet*, *decides on own health and diet during pregnancy* and *decides on weaning and breastfeeding*. In other words, women who have more access to food and health products may not necessarily have input into decisions on their own health and diet, breastfeeding and weaning, suggesting that in better‐off households, women's agency over their own health may still be constrained. Additionally, redundancy is also low between *decides on own health and diet* and *decides on child's diet*.

### 3.6 Associations with pro‐WEAI indicators

Across all projects, the magnitude of associations between the seven health and nutrition indicators and 12 pro‐WEAI indicators were generally low (V <0.30) (Supporting Information: Appendix Table 14). The associations were, however, higher for sets of similar indicators. *Productive decisions* is based on survey items with a similar structure to the decision in the health and nutrition module. Additionally, *decides to purchase food and health products* is more strongly associated with *access to and decisions on financial services* and *control over use of income*, which are all linked to control of money. This finding suggests that the new health and nutrition indicators measure something that is not being measured by the core pro‐WEAI and points to their added value.

### 3.7 Correlates of empowerment

Mean levels of adequacy varied by women's age group for all seven indicators in Bangladesh and four of them in the pooled Burkina Faso‐Mali sample (Supporting Information: Appendix Figure 2). In Bangladesh, the 25−34‐year‐olds were consistently more empowered than the 16−24‐year‐olds. This was not the case for women older than 35, which may be attributable to the rapid expansion of women's education between these two cohorts or because older mothers are select high‐parity women. In Burkina Faso and Mali, the age‐related patterns were not as strong. Additionally, the oldest groups often had fewer women achieving adequacy, likely for similar reasons suggested for the Bangladesh results.

Mean levels of adequacy differed by educational attainment for five of the seven indicators in Bangladesh and two of the indicators in the Burkina Faso‐Mali samples (Supporting Information: Appendix Figure A3). Overall, the differences by educational attainment did not reveal a strong pattern of higher levels of adequacy for higher educational attainment, but most of the significant differences were in the hypothesized direction, except for one case where the magnitude is small. The weak patterns between educational attainment and adequacy may again be the result of rapid educational expansion.

## 4 DISCUSSION

We describe the development and validation of a survey module and indicators for measuring women's instrumental agency in health and nutrition, which are designed to complement core pro‐WEAI (H. Malapit et al., 2019). Using data from Bangladesh, Burkina Faso and Mali, we developed seven indicators: *decides on own health and diet*, *decides on health and diet during pregnancy*, *decides on child's diet*, *decides on weaning and breastfeeding*, *decides to seek healthcare*, *decides to purchase food and health products* and *has access to food and health products*. They are based on factors that meet standards of acceptable fit and measurement invariance across different contexts and data collection firms within similar areas. According to tests of association and redundancy, they measure dimensions of agency that are distinct from those measured by core pro‐WEAI and address different processes in the pathways between agriculture and nutrition (Gillespie et al., 2012; Kadiyala et al., 2014).

The pro‐WEAI + HN meets the need for a standardized women's empowerment metric that measures multiple domains of empowerment important for health and nutrition outcomes (Santoso et al., 2019). It was designed for and validated in multiple country contexts, for six different projects, five different data collection entities and over 12,000 individuals. Additionally, it was designed for nutrition‐sensitive agriculture programmes by considering the programme impact pathways and nutritionally vulnerable periods during the questionnaire design (Gillespie et al., 2012; Kadiyala et al., 2014; Ruel & Alderman, 2013). Comparing pro‐WEAI + HN to other approaches, the WENI, for example, focused on nutrition‐related empowerment but did not address animal‐source foods, allocation within the home or child nutrition as the pro‐WEAI + HN does (Narayanan et al., 2019). Pro‐WEAI + HN also improves on metrics designed for a single context, as it allows for comparisons across contexts.

This module provides insights beyond core pro‐WEAI alone by looking beyond productive domains. Pro‐WEAI + HN can help test important hypotheses about the nature of women's empowerment and the aspects that are important for improving diets and nutritional status when collected alongside data on these other outcomes. Until now, it has not been possible to evaluate whether women's empowerment in the productive sphere, domestic sphere or a combination of both leads to improved nutrition and health outcomes. Such findings are important for determining how to strategically prioritize interventions.

### 4.1 Limitations and future research

Several limitations are worth noting. The findings are based on one round of data collection for each project and a limited number of settings. Additionally we did not have data that would have allowed us to further assess construct and criterion validity. Future work should examine how these indicators perform over time, including evaluating measurement equivalence, sensitivity to programme impact and associations with other outcomes. We also encourage additional studies that examine the associations between these indicators and specific expected nutrition and health outcomes to provide further evidence of validity. For example, studies could examine whether decisions on maternal and child diets are associated with higher dietary diversity and whether decisions about healthcare are associated with healthcare utilization. Additionally, these instruments and indicators should be tested in other contexts to ensure their validity more broadly. Another shortcoming is the lack of attention to agency in water, sanitation and hygiene (WASH), which also influence nutritional outcomes. The current module was developed with projects that did not have a WASH focus.

Additionally, the pro‐WEAI + HN was designed for and fielded in the context of gender‐ and nutrition‐sensitive agricultural development projects. Although some of the indicators may be appropriate for nutrition‐specific and other types of nutrition‐sensitive programmes, we do not yet have this evidence but encourage others to consider these indicators.

### 4.2 Conclusion: Using the pro‐WEAI + HN

To conclude, we provide suggestions for incorporating these indicators into impact evaluations. Firstly, the HN add‐on is designed to be administered in addition to core pro‐WEAI. The association and redundancy findings suggest that HN indicators will allow studies to assess dimensions of women's empowerment beyond the productive dimensions currently measured by the core pro‐WEAI. For the seven HN indicators, studies should use the individual indicators and not aggregate them, which would ignore the multidimensionality we identified. Additionally, some indicators apply only to women in particular lifecycle phases (e.g., pregnancy) and should only be calculated or interpreted for those women.

Additionally, pro‐WEAI + HN is compatible with common impact assessment designs, and special considerations should be made for the lifecycle‐specific indicators. Impact assessments of nutrition‐sensitive agriculture programmes that target children or pregnant women typically use (1) repeated cross‐sectional surveys that select households with children in a target age range or (2) panel surveys of households with young children or pregnant women. For repeated cross‐sectional surveys, it is possible to compare the indicators at baseline and follow‐up using cluster fixed‐effects double‐difference models for the subsamples for which the indicator is applicable. For panel surveys, which often collect different lifecycle‐related indicators at baseline and follow‐up, one can estimate the impact on an outcome, such as *decides on child diet*, within the subpopulation, using an analysis of covariance model that controls for related baseline characteristics, which may include different pro‐WEAI + HN indicators from an earlier wave, such as *decides on health and diet during pregnancy*. Moreover, it is important to consider the size of the subpopulation when conducting power calculations.

Overall, the pro‐WEAI + HN, has the potential to strengthen the evidence on how nutrition‐sensitive agriculture programmes can increase women's empowerment and the extent to which women's empowerment in the productive and domestic spheres can contribute to other nutrition and health outcomes for women and their family members. This empirical evidence can, in turn, help prioritize interventions with the greatest potential, ultimately improving both empowerment‐ and nutrition‐related outcomes.

## AUTHOR CONTRIBUTIONS

*Conceptualization*: Jessica Heckert, Sunny S. Kim, Hazel Malapit, Greg Seymour and Shalini Roy. *Methodology*: Jessica Heckert, Elena M. Martinez and Greg Seymour. *Formal analysis*: Jessica Heckert, Elena M. Martinez, Greg Seymour and Audrey Pereira. *Data Curation*: Elena M. Martinez and Audrey Pereira. *Writing—original draft*: Jessica Heckert, Elena M. Martinez and Audrey Pereira. *Writing—reviewing and editing*: Jessica Heckert, Sunny S. Kim, Hazel Malapit, Elena M. Martinez, Audrey Pereira, Shalini Roy and Greg Seymour. *Supervision*: Jessica Heckert and Hazel Malapit.

## GENDER ASSETS AND AGRICULTURE PROJECT PHASE 2 (GAAP2) HEALTH AND NUTRITION STUDY TEAM

Gender, Agriculture, and Assets Project Phase Two (GAAP2) Principal Investigators: Agnes Quisumbing, Ruth Meinzen‐Dick and Hazel Malapit; study team of Agriculture, Nutrition and Gender Linkages (ANGeL): Akhter Ahmed, Anika Hannan, Shalini Roy, Masuma Younus; study team of Building resilience of vulnerable communities in Burkina Faso (Grameen): Benjamin Crookston, Megan Gash, Bobbi Gray; study team of Deploying improved vegetable technologies to overcome malnutrition and poverty (WorldVeg): Pepijn Schreinemachers and Caroline Sobgui; study team of Food and Agricultural Approaches to Reducing Malnutrition (FAARM): Sabine Gabrysch, Sheela Sinharoy, Jillian Waid and Amanda Wendt; study team of Integrated poultry value chain and nutrition intervention (SELEVER): Josué Awonon, Rasmané Ganaba, Aulo Gelli, Abdoulaye Pedehombga, Armande Sanou, Sita Zougouri; and study team of Targeting and Realigning Agriculture to Improve Nutrition (TRAIN): Neha Kumar, Mamun Miah, Saiqa Siraj.

## CONFLICT OF INTEREST

The authors declare no conflict of interest.

## Supporting information

Supporting information.

## Data Availability

The data that support the findings will be available publicly following an embargo from the date of publication to allow for the production of additional research findings.
