# Supplementary material for: Development and validation of a health and nutrition module for the project‐level Women's Empowerment in Agriculture Index (pro‐WEAI+HN)
Source: Matern Child Nutr. 2022 Dec 8;19(2):e13464. doi: 10.1111/mcn.13464 (PMC10019063; doi:10.1111/mcn.13464)
Supplement: Supplementary file 1 — Supporting information. [file MCN-19-e13464-s001.docx]

**Supplementary Appendix**

**Appendix Table A1.** Survey items in the pro-WEAI health and nutrition modules

| **DECISIONS ABOUT WOMEN'S HEALTH AND NUTRITION** | | |
| --- | --- | --- |
| **Questions** | **Activities** | **Response options** |
| **Q1.** When decisions are made about [ACTIVITY], who normally takes the decision?  **Q2.** To what extent do you participate in decisions regarding [ACTIVITY]? | 1. Whether or not you consult a doctor or go to a clinic when you are ill B. How much you can rest when you are ill C. Whether or not you have a/another child † D. Whether or not you use a contraceptive method (such as birth control pills, condoms, hormonal shot, or sterilization)? † E. What foods to prepare every day F. What foods (available in the house) you can eat G. Whether you consulted a doctor or went to a clinic during your current or most recent pregnancy* H. How much you worked during your current or most recent pregnancy* I. How much you could rest during your current or most recent pregnancy* J. Whether you could eat eggs during your current or most recent pregnancy* K. Whether you could consume milk or milk products during your current or most recent pregnancy* L. Whether you could eat meat, poultry, or fish during your current or most recent pregnancy* M. How much you worked when your youngest child was being breastfed* † N. How much you could rest when your youngest child was being breastfed* † O. Whether you could eat eggs when your youngest child was being breastfed* † P. Whether you could consume milk or milk products when your youngest child was being breastfed* † Q. Whether you could eat meat, poultry, or fish when your youngest child was being breastfed* † | **Q1.** Enter up to three member IDs  (linked to names in household roster via a computer-assisted personal interviewing)  **Q2.** (Select one)  Not at all (1)  To a small extent (2)  To a medium extent (3)  To a high extent (4)  (linked to names in household roster via a computer-assisted personal interviewing) |
| **DECISIONS ABOUT CHILD HEALTH AND NUTRITION** | | |
| **Questions** | **Activities** | **Response options** |
| **Q3.** When decisions are made about [ACTIVITY], who normally takes the decision?  **Q4.** To what extent do you participate in decisions regarding [ACTIVITY]? | A. Whether your child is taken to a clinic or a doctor is consulted when he/she is sick B. Whether your child gets vaccinations † C. Whether your child visits the health clinic to see if he/she is growing well  D. How to feed your child when he/she is sick † E. Who will care for your child when you need to go outside the home for an extended period of time † F. Sending your child to school † G. Whether your child is offered eggs to eat** H. Whether your child is offered milk or milk products, other than breastmilk** I. Whether your child is offered meat, poultry, or fish** J. Whether to breastfeed your child*** K. When to stop breastfeeding your child*** L. When to start introducing foods and liquids (other than breastmilk) to your child M. Whether or not your child is fed foods prepared or bought especially for children that adult household members do not eat or drink, such as fortified cereals or baby foods? ** *** † | **Q1.** Enter up to three member IDs (linked to names in household roster via a computer-assisted personal interviewing)  **Q2.** (Select one)  Not at all (1)  To a small extent (2)  To a medium extent (3)  To a high extent (4) |
| **HEALTH AND NUTRITION PRODUCTS** | | |
| **Questions** | **Products** | **Response options** |
| **Q5**. When decisions are made whether or not to purchase [PRODUCT], who generally makes the decision?  **Q6.** You may acquire an item that you need in a variety of ways, such as purchasing or cultivating it or having someone purchase or cultivate it for you. When you need [PRODUCT], can you usually acquire it? | A. Small quantities of food, for example smaller than 5 kg B. Large quantities of food, for example larger than 5 kg C. Eggs D. Milk or milk products E. Meat, poultry, or fish (including organ meats) F. Special foods for children (i.e., foods prepared or bought especially for children that adult household members do not eat or drink, such as fortified cereals or baby foods that programs or health workers tell you should be consumed † G. Any nutritious foods that a program or health worker told you to consume † H. Medication, vitamins, or supplements for children I. Medication, vitamins, or supplements for yourself J. Clothing for children † K. Clothing for yourself † L. Toiletries, such as soap and toothpaste | **Q1.** Enter up to three member IDs  (linked to names in household roster via a computer-assisted personal interviewing)  **Q2.** (Select one)  Yes  No  Not applicable |
| * Only asked to women who have been pregnant or given birth in the last 2 years | |  |
| ** Only asked to women who have a child aged 6 months or older | |  |
| *** Only asked to women who have a child aged 2 years or younger | |  |
| † Item not used in indicator construction | |  |

**Appendix Table A2.** Description of data sources

| Project name | Geography | Partners | Focus | Year data collected | Sample selection | Sample size^1^ | Key citation | Ethics Approval (entity and study number) |
| --- | --- | --- | --- | --- | --- | --- | --- | --- |
| Agriculture, Nutrition, and Gender Linkages (ANGeL) | Bangladesh | Ministry of Agriculture (Bangladesh) and IFPRI | delivered a program to households that focused on diversifying agricultural production, increasing nutrition knowledge, and enhancing gender sensitization | 2018 | farming households with at least one child younger than 24 months | Total=3,917  Pregnant<2 years=491  Child < 2 years=392 | (Ahmed et al. 2017) | IRB of IFPRI, 2016-8-PHND-M |
| Food and Agricultural Approaches to Reducing Malnutrition, (FAARM) | Bangladesh | Heidelberg University and Helen Keller International | enhancing homestead food production by training rural women’s groups in vegetable, fruit, and poultry production, nutrition, and hygiene | 2016 | married women aged 15-30 years | Total=287  Pregnant<2 years=138  Child < 2 years=92  *Omitted 15 items | (Wendt et al., 2019) | Ethical Review Committee, Jams P Grant School of Public Health at BRAC University, 37A |
| Building Resilience of Vulnerable Communities, in Burkina Faso (Grameen) | Burkina Faso | Grameen Foundation and Brigham Young University | used savings groups to deliver a package of interventions around agriculture, nutrition, financial services, and women’s empowerment | 2016 | women who were members of savings groups | Total=380  Pregnant<2 years=83  Child < 2 years=87 | (Gash and Gray 2016) | IRB of Brigham Young University, X16090 |
| Soutenir l’Exploitation Famaliales pour Lancer l’Elevage des Volailles et Valoriser l’Economie Rurale (SELEVER) | Burkina Faso | Agribusiness Systems International, AfricSante, and IFPRI | used a poultry value chain project as a platform for integrating nutrition- and gender-sensitive programming | 2018 | households with women aged 15-35 years and at least one child aged 2-4 years | Total=1,777  Pregnant<2 years=816  Child < 2 years=633 | (Gelli et al., 2017) | Comite d’éthique pour la Recherche en Santé MS/MRSI in Burkina Faso (2016-12-142); IRB of IFPRI, IRB00007490 |
| Targeting and Realigning Agriculture to Improve Nutrition (TRAIN), | Bangladesh | BRAC and IFPRI | increase agricultural production and diet diversity by linking to an existing agricultural credit program | 2016-17 | targeted households where women were likely to become pregnant in the near future, based on recent marriage and not yet having two children | Total=5,039  Pregnant<2 years=2,253  Child < 2 years=1,426 | (Kumar & Ruel, 2020) | IRB of IFPRI, 2016-24-PHND-M |
| Deploying Improved Vegetable Technologies to Overcome Malnutrition and Poverty | Mali | World Vegetable Center | engage women in organized platforms that connect them to improved vegetable technologies | 2016 | households with women aged 15-49 years and oversampled households with a child younger than five years old | Total=714  Pregnant<2 years=389  Child < 2 years=431 | (Schreinemachers et al., 2016) | Comite d’Ethique de la Faculte de Medecine de Pharmacie et d’Odonto-Stomatologie, L’Universite des Science des Techniques et des Technologies de Bamako |

Note: FAARM omitted 15 items, which prevented us from including it in some parts of the analysis, but we calculate the indicators when possible. SELEVER omitted items on purchasing/accessing medications or vitamins for self; we are unable to include this item in the EFA and CFA and treat it as non-applicable when we calculate the indicators. Item non-response for applicable items was non-existent for Grameen and TRAIN; negligible for AGEeL (n<=2); and low for FAARM (n=79 for visits to health clinics, n<=15 for all other items) and WorldVeg (n=23/20 for breastfeeding/weaning items, n <=5 for all other items). SELEVER had a higher rate of item non-response, as high as 455 for one item, primarily attributable to a data collection programing error.

**Appendix Table A3.** Means of survey items used in exploratory and confirmatory factor analyses of women’s participation in decisions related to women’s and children’s health by project

|  | All projects | | ANGeL (CFA) | | FAARM (CFA) | | Grameen (CFA) | | SELEVER (EFA) | | SELEVER (CFA) | | TRAIN (EFA) | | TRAIN (CFA) | | WorldVeg (CFA) | |
| --- | --- | --- | --- | --- | --- | --- | --- | --- | --- | --- | --- | --- | --- | --- | --- | --- | --- | --- |
| **Survey item** | **Mean** | **SE** | **Mean** | **SE** | **Mean** | **SE** | **Mean** | **SE** | **Mean** | **SE** | **Mean** | **SE** | **Mean** | **SE** | **Mean** | **SE** | **Mean** | **SE** |
| Rest when ill | 3.46 | 0.02 | 3.75 | 0.03 | 3.74 | 0.07 | 2.96 | 0.16 | 3.56 | 0.05 | 3.58 | 0.04 | 3.57 | 0.03 | 3.59 | 0.03 | 3.29 | 0.05 |
| Foods to prepare | 3.46 | 0.02 | 3.46 | 0.04 | 3.86 | 0.06 | 3.26 | 0.16 | 3.44 | 0.05 | 3.50 | 0.04 | 3.68 | 0.03 | 3.64 | 0.03 | 3.38 | 0.04 |
| Foods to eat | 3.41 | 0.02 | 3.48 | 0.04 | 3.96 | 0.03 | 3.15 | 0.16 | 3.19 | 0.06 | 3.38 | 0.05 | 3.82 | 0.02 | 3.81 | 0.02 | 3.31 | 0.05 |
| Work during pregnancy | 3.44 | 0.02 | 3.66 | 0.04 | -- | -- | 3.24 | 0.14 | 3.58 | 0.04 | 3.65 | 0.04 | 3.55 | 0.03 | 3.54 | 0.03 | 3.25 | 0.05 |
| Rest during pregnancy | 3.50 | 0.02 | 3.71 | 0.03 | -- | -- | 3.17 | 0.16 | 3.59 | 0.04 | 3.69 | 0.04 | 3.67 | 0.03 | 3.69 | 0.03 | 3.30 | 0.05 |
| Eggs during pregnancy | 3.41 | 0.02 | 3.73 | 0.03 | 3.84 | 0.08 | 3.09 | 0.16 | 3.41 | 0.05 | 3.45 | 0.05 | 3.65 | 0.03 | 3.63 | 0.03 | 3.24 | 0.05 |
| Milk or milk products during pregnancy | 3.40 | 0.02 | 3.73 | 0.03 | -- | -- | 3.13 | 0.15 | 3.39 | 0.05 | 3.47 | 0.05 | 3.63 | 0.03 | 3.61 | 0.03 | 3.22 | 0.05 |
| Meat, poultry, or fish during pregnancy | 3.39 | 0.02 | 3.74 | 0.03 | -- | -- | 3.33 | 0.14 | 3.41 | 0.05 | 3.48 | 0.04 | 3.58 | 0.03 | 3.53 | 0.03 | 3.22 | 0.05 |
| Work during breastfeeding† | 3.52 | 0.02 | 3.78 | 0.03 | 3.96 | 0.03 | 3.20 | 0.17 | 3.57 | 0.04 | 3.68 | 0.04 | 3.74 | 0.03 | 3.68 | 0.03 | 3.32 | 0.05 |
| Rest during breastfeeding† | 3.55 | 0.02 | 3.77 | 0.03 | -- | -- | 3.26 | 0.16 | 3.59 | 0.04 | 3.71 | 0.04 | 3.76 | 0.03 | 3.75 | 0.03 | 3.37 | 0.05 |
| Eggs during breastfeeding† | 3.45 | 0.02 | 3.77 | 0.03 | -- | -- | 3.43 | 0.13 | 3.39 | 0.05 | 3.48 | 0.05 | 3.73 | 0.03 | 3.69 | 0.03 | 3.30 | 0.05 |
| Milk or milk products during breastfeeding† | 3.47 | 0.02 | 3.76 | 0.03 | -- | -- | 3.48 | 0.12 | 3.40 | 0.05 | 3.51 | 0.04 | 3.74 | 0.03 | 3.68 | 0.03 | 3.33 | 0.05 |
| Meat, poultry, or fish during breastfeeding† | 3.43 | 0.02 | 3.73 | 0.03 | -- | -- | 3.33 | 0.14 | 3.41 | 0.05 | 3.46 | 0.05 | 3.68 | 0.03 | 3.63 | 0.03 | 3.28 | 0.05 |
| Child vaccinated† | 3.52 | 0.02 | 3.66 | 0.03 | -- | -- | 2.85 | 0.18 | 3.59 | 0.04 | 3.62 | 0.04 | 3.74 | 0.03 | 3.76 | 0.03 | 3.35 | 0.04 |
| Feed child when sick† | 3.57 | 0.02 | 3.70 | 0.03 | 3.94 | 0.03 | 3.35 | 0.14 | 3.67 | 0.04 | 3.70 | 0.04 | 3.73 | 0.03 | 3.71 | 0.03 | 3.42 | 0.04 |
| Whether to breastfeed | 3.80 | 0.01 | 3.75 | 0.03 | -- | -- | 3.96 | 0.03 | 3.81 | 0.03 | 3.86 | 0.03 | 3.89 | 0.02 | 3.89 | 0.02 | 3.76 | 0.03 |
| When to stop breastfeeding | 3.81 | 0.01 | 3.90 | 0.02 | -- | -- | 3.89 | 0.05 | 3.80 | 0.04 | 3.87 | 0.03 | 3.88 | 0.02 | 3.90 | 0.02 | 3.75 | 0.03 |
| Introducing foods and liquids | 3.74 | 0.02 | 3.88 | 0.02 | 3.92 | 0.04 | 3.67 | 0.11 | 3.80 | 0.03 | 3.86 | 0.03 | 3.86 | 0.02 | 3.82 | 0.02 | 3.61 | 0.04 |
| Child is fed special foods for children† | 3.63 | 0.02 | 3.82 | 0.03 | -- | -- | 3.50 | 0.14 | 3.61 | 0.05 | 3.60 | 0.05 | 3.71 | 0.03 | 3.71 | 0.03 | 3.59 | 0.04 |
| Child is offered eggs | 3.47 | 0.02 | 3.77 | 0.03 | 3.84 | 0.07 | 3.22 | 0.17 | 3.31 | 0.06 | 3.41 | 0.05 | 3.77 | 0.02 | 3.74 | 0.03 | 3.38 | 0.04 |
| Child is offered milk or milk products | 3.50 | 0.02 | 3.78 | 0.03 | -- | -- | 3.28 | 0.16 | 3.40 | 0.06 | 3.50 | 0.05 | 3.77 | 0.03 | 3.75 | 0.03 | 3.38 | 0.04 |
| Child is offered meat, poultry, or fish | 3.49 | 0.02 | 3.77 | 0.03 | 3.68 | 0.09 | 3.37 | 0.15 | 3.41 | 0.05 | 3.47 | 0.05 | 3.75 | 0.03 | 3.72 | 0.03 | 3.37 | 0.04 |
| Go to doctor when ill | 3.17 | 0.02 | 3.31 | 0.04 | 3.14 | 0.14 | 2.87 | 0.17 | 3.03 | 0.05 | 3.20 | 0.05 | 3.21 | 0.04 | 3.23 | 0.04 | 3.17 | 0.05 |
| Have another child† | 3.25 | 0.03 | 3.74 | 0.03 | -- | -- | 2.80 | 0.18 | 2.91 | 0.06 | 2.93 | 0.06 | 3.43 | 0.04 | 3.48 | 0.04 | 3.31 | 0.05 |
| Use contraceptives† | 3.19 | 0.03 | 3.82 | 0.03 | 3.08 | 0.15 | 2.67 | 0.18 | 2.70 | 0.07 | 2.70 | 0.07 | 3.48 | 0.04 | 3.52 | 0.04 | 3.28 | 0.05 |
| Go to doctor during pregnancy | 3.32 | 0.02 | 3.44 | 0.04 | 3.40 | 0.12 | 3.02 | 0.16 | 3.27 | 0.05 | 3.44 | 0.04 | 3.35 | 0.04 | 3.38 | 0.04 | 3.26 | 0.04 |
| Whether to take sick child to doctor | 3.29 | 0.02 | 3.42 | 0.04 | 3.70 | 0.09 | 2.67 | 0.19 | 3.16 | 0.05 | 3.17 | 0.05 | 3.58 | 0.03 | 3.58 | 0.03 | 3.24 | 0.05 |
| Whether to take child for well visits | 3.44 | 0.02 | 3.53 | 0.04 | 3.54 | 0.13 | 3.04 | 0.17 | 3.56 | 0.04 | 3.54 | 0.04 | 3.61 | 0.03 | 3.64 | 0.03 | 3.27 | 0.05 |
| Who cares for child when you are outside the home† | 3.52 | 0.02 | 3.56 | 0.04 | -- | -- | 3.24 | 0.17 | 3.60 | 0.04 | 3.59 | 0.04 | 3.51 | 0.03 | 3.56 | 0.03 | 3.47 | 0.04 |
| **Source:** Project data from ANGeL (N=3,917), FAARM (N=287), Grameen (N=380), SELEVER (N=1,777), TRAIN (N=5,040), and WorldVeg (N=714) | | | | | | | | | | | | | | | | | | |
| **Note:** Means for all projects combined are weighted by the inverse of project sample size, so that characteristics are equally weighted by project sample. Response categories for survey items on women’s participation in decisions related to women’s and child health and nutrition are: 1=feels cannot participate in (joint) decision at all, 2=feels can participate in (joint) decisions to a small extent, 3=feels can participate in (joint) decisions to a medium extent, and 4=sole decision-maker or feels can participate in (joint) decision to a high extent. FAARM omitted some survey items from their survey. EFAs were conducted using a random half of the data from SELEVER and TRAIN; CFAs were conducted using the other random half of the data from SELEVER and TRAIN, as well as data from ANGeL, FAARM, Grameen, and WorldVeg.  † Item not used in indicator construction | | | | | | | | | | | | | | | | | | |

**Appendix Table A4.** Means of survey items used in exploratory and confirmatory factor analysis of women’s participation in decisions about purchasing and the ability to acquire health products related to women’s and children’s health by project.

|  | All projects | | | ANGeL | | | | FAARM | | | | Grameen | | | | SELEVER (EFA) | | | | SELEVER (CFA) | | | | TRAIN (EFA) | | | | TRAIN (CFA) | | | | WorldVeg | | | | |  |  |
| --- | --- | --- | --- | --- | --- | --- | --- | --- | --- | --- | --- | --- | --- | --- | --- | --- | --- | --- | --- | --- | --- | --- | --- | --- | --- | --- | --- | --- | --- | --- | --- | --- | --- | --- | --- | --- | --- | --- |
| **Survey item** | **Mean** | | **SE** | | **Mean** | | **SE** | | **Mean** | | **SE** | | **Mean** | | **SE** | | **Mean** | | **SE** | | **Mean** | | **SE** | | **Mean** | | **SE** | | **Mean** | | **SE** | | | **Mean** | | **SE** | | |
| Respondent contributes to the decision to purchase: | | | | | | | | | | | | | | | | | | | | | | | | | | | | | | | | | | | | | |  |
| Small foods | 0.68 | 0.01 | | 0.74 | | 0.01 | | 0.90 | | 0.02 | | 0.57 | | 0.03 | | 0.29 | | 0.02 | | 0.29 | | 0.02 | | 0.72 | | 0.01 | | 0.72 | | 0.01 | | 0.74 | | | 0.02 | |  |  |
| Large foods | 0.60 | 0.01 | | 0.71 | | 0.01 | | 0.79 | | 0.03 | | 0.38 | | 0.03 | | 0.20 | | 0.02 | | 0.20 | | 0.02 | | 0.68 | | 0.01 | | 0.67 | | 0.01 | | 0.70 | | | 0.02 | |  |  |
| Eggs | 0.72 | 0.01 | | 0.83 | | 0.01 | | 0.93 | | 0.02 | | 0.57 | | 0.03 | | 0.28 | | 0.02 | | 0.30 | | 0.02 | | 0.75 | | 0.01 | | 0.77 | | 0.01 | | 0.77 | | | 0.02 | |  |  |
| Milk | 0.76 | 0.01 | | 0.82 | | 0.01 | | -- | | -- | | 0.73 | | 0.02 | | 0.32 | | 0.02 | | 0.33 | | 0.02 | | 0.75 | | 0.01 | | 0.76 | | 0.01 | | 0.76 | | | 0.02 | |  |  |
| Meat/poultry/fish | 0.68 | 0.01 | | 0.80 | | 0.01 | | 0.84 | | 0.02 | | 0.58 | | 0.03 | | 0.27 | | 0.02 | | 0.25 | | 0.02 | | 0.72 | | 0.01 | | 0.74 | | 0.01 | | 0.73 | | | 0.02 | |  |  |
| Special foods for children† | 0.78 | 0.01 | | 0.89 | | 0.01 | | -- | | -- | | 0.67 | | 0.02 | | 0.35 | | 0.02 | | 0.34 | | 0.02 | | 0.82 | | 0.01 | | 0.85 | | 0.01 | | 0.79 | | | 0.02 | |  |  |
| Nutritious program foods† | 0.76 | 0.01 | | 0.90 | | 0.01 | | - | | -- | | 0.62 | | 0.03 | | 0.37 | | 0.02 | | 0.37 | | 0.02 | | 0.83 | | 0.01 | | 0.85 | | 0.01 | | 0.74 | | | 0.02 | |  |  |
| Medicine for child | 0.74 | 0.01 | | 0.88 | | 0.01 | | 0.94 | | 0.01 | | 0.58 | | 0.03 | | 0.32 | | 0.02 | | 0.31 | | 0.02 | | 0.83 | | 0.01 | | 0.86 | | 0.01 | | 0.73 | | | 0.02 | |  |  |
| Medicine for self | 0.82 | 0.01 | | 0.88 | | 0.01 | | 0.93 | | 0.02 | | 0.65 | | 0.02 | | -- | | -- | | -- | | -- | | 0.82 | | 0.01 | | 0.84 | | 0.01 | | 0.73 | | | 0.02 | |  |  |
| Clothing for child† | 0.82 | 0.01 | | 0.87 | | 0.01 | | -- | | -- | | 0.88 | | 0.02 | | 0.46 | | 0.02 | | 0.47 | | 0.02 | | 0.84 | | 0.01 | | 0.84 | | 0.01 | | 0.74 | | | 0.02 | |  |  |
| Clothing for self† | 0.81 | 0.01 | | 0.87 | | 0.01 | | 0.87 | | 0.02 | | 0.89 | | 0.02 | | 0.54 | | 0.02 | | 0.56 | | 0.02 | | 0.82 | | 0.01 | | 0.83 | | 0.01 | | 0.76 | | | 0.02 | |  |  |
| Toiletries | 0.82 | 0.01 | | 0.84 | | 0.01 | | 0.91 | | 0.02 | | 0.93 | | 0.01 | | 0.51 | | 0.02 | | 0.52 | | 0.02 | | 0.79 | | 0.01 | | 0.80 | | 0.01 | | 0.79 | | | 0.02 | |  |  |
| Respondent is typically able to acquire the product by some means: | | | | | | | | | | | | | | | | | | | | | | | | | | | | | | | | | | | | | |  |
| Small foods | 0.90 | 0.01 | | 0.94 | | 0.00 | | 0.94 | | 0.01 | | 0.89 | | 0.02 | | 0.90 | | 0.01 | | 0.92 | | 0.01 | | 0.87 | | 0.01 | | 0.87 | | 0.01 | | 0.86 | | | 0.01 | |  |  |
| Large foods | 0.78 | 0.01 | | 0.86 | | 0.01 | | 0.63 | | 0.03 | | 0.79 | | 0.02 | | 0.78 | | 0.02 | | 0.79 | | 0.02 | | 0.80 | | 0.01 | | 0.82 | | 0.01 | | 0.80 | | | 0.02 | |  |  |
| Eggs | 0.90 | 0.01 | | 0.98 | | 0.00 | | 0.96 | | 0.01 | | 0.85 | | 0.02 | | 0.83 | | 0.02 | | 0.85 | | 0.02 | | 0.90 | | 0.01 | | 0.90 | | 0.01 | | 0.88 | | | 0.01 | |  |  |
| Milk | 0.91 | 0.00 | | 0.97 | | 0.00 | | -- | | -- | | 0.89 | | 0.02 | | 0.82 | | 0.02 | | 0.86 | | 0.02 | | 0.90 | | 0.01 | | 0.89 | | 0.01 | | 0.86 | | | 0.01 | |  |  |
| Meat/poultry/fish | 0.86 | 0.01 | | 0.94 | | 0.00 | | 0.79 | | 0.03 | | 0.92 | | 0.01 | | 0.77 | | 0.02 | | 0.81 | | 0.02 | | 0.85 | | 0.01 | | 0.84 | | 0.01 | | 0.83 | | | 0.01 | |  |  |
| Special foods for children† | 0.86 | 0.01 | | 0.91 | | 0.00 | | -- | | -- | | 0.70 | | 0.02 | | 0.72 | | 0.02 | | 0.74 | | 0.02 | | 0.86 | | 0.01 | | 0.86 | | 0.01 | | 0.93 | | | 0.01 | |  |  |
| Nutritious program foods† | 0.84 | 0.01 | | 0.92 | | 0.00 | | -- | | -- | | 0.70 | | 0.02 | | 0.71 | | 0.02 | | 0.74 | | 0.02 | | 0.83 | | 0.01 | | 0.83 | | 0.01 | | 0.82 | | | 0.01 | |  |  |
| Medicine for child | 0.85 | 0.01 | | 0.93 | | 0.00 | | 0.90 | | 0.02 | | 0.74 | | 0.02 | | 0.82 | | 0.02 | | 0.86 | | 0.02 | | 0.87 | | 0.01 | | 0.86 | | 0.01 | | 0.82 | | | 0.01 | |  |  |
| Medicine for self | 0.86 | 0.01 | | 0.93 | | 0.00 | | 0.86 | | 0.02 | | 0.75 | | 0.02 | | -- | | -- | | -- | | -- | | 0.85 | | 0.01 | | 0.85 | | 0.01 | | 0.85 | | | 0.01 | |  |  |
| Clothing for child† | 0.92 | 0.00 | | 0.92 | | 0.00 | | -- | | -- | | 0.95 | | 0.01 | | 0.93 | | 0.01 | | 0.95 | | 0.01 | | 0.87 | | 0.01 | | 0.87 | | 0.01 | | 0.86 | | | 0.01 | |  |  |
| Clothing for self† | 0.88 | 0.01 | | 0.91 | | 0.00 | | 0.74 | | 0.03 | | 0.96 | | 0.01 | | 0.93 | | 0.01 | | 0.96 | | 0.01 | | 0.84 | | 0.01 | | 0.85 | | 0.01 | | 0.90 | | | 0.01 | |  |  |
| Toiletries | 0.92 | 0.00 | | 0.95 | | 0.00 | | 0.88 | | 0.02 | | 0.97 | | 0.01 | | 0.90 | | 0.01 | | 0.93 | | 0.01 | | 0.88 | | 0.01 | | 0.87 | | 0.01 | | 0.93 | | | 0.01 | |  |  |
| **Source:** Project data from ANGeL (N=3,917), FAARM (N=287), Grameen (N=380), SELEVER (N=1,777), TRAIN (N=5,040), and WorldVeg (N=714) | | | | | | | | | | | | | | | | | | | | | | | | | | | | | | | | |  |  |  |  |  |  |
| **Note**: Means for all projects combined are weighted by the inverse of project sample size, so that characteristics are equally weighted by project sample. FAARM and SELEVER omitted some survey items from their surveys. EFAs were conducted using a random half of the data from SELEVER and TRAIN; CFAs were conducted using the other random half of the data from SELEVER and TRAIN, as well as data from ANGeL, FAARM, Grameen, and WorldVeg.  † Item not used in indicator construction | | | | | | | | | | | | | | | | | | | | | | | | | | | | | | | | |  |  |  |  |  |  |

**Appendix Table A5.** Pairwise correlations between survey items included in the exploratory factor analysis.

*Source: Responses of women from dual-adult households who had been pregnant or given birth in the last two years and had a child under age two, from a random half sample of data from the SELEVER and TRAIN projects*

|  | **Survey item** | **1** | **2** | **3** | **4** | **5** | **6** | **7** | **8** | **9** | **10** |
| --- | --- | --- | --- | --- | --- | --- | --- | --- | --- | --- | --- |
| **1** | Rest when ill | 1.00 |  |  |  |  |  |  |  |  |  |
| **2** | Foods to prepare | .49 | 1.00 |  |  |  |  |  |  |  |  |
| **3** | Foods you can eat | .41 | .58 | 1.00 |  |  |  |  |  |  |  |
| **4** | Work during pregnancy | .56 | .45 | .28 | 1.00 |  |  |  |  |  |  |
| **5** | Rest during pregnancy | .62 | .47 | .36 | .75 | 1.00 |  |  |  |  |  |
| **6** | Eat eggs during pregnancy | .51 | .43 | .39 | .58 | .69 | 1.00 |  |  |  |  |
| **7** | Consume milk or milk products during pregnancy | .49 | .45 | .39 | .57 | .64 | .80 | 1.00 |  |  |  |
| **8** | Eat meat, poultry, or fish during pregnancy | .50 | .44 | .38 | .58 | .64 | .74 | .79 | 1.00 |  |  |
| **9** | Work during breastfeeding† | .58 | .48 | .37 | .63 | .69 | .68 | .69 | .67 | 1.00 |  |
| **10** | Rest during breastfeeding† | .54 | .44 | .35 | .57 | .67 | .62 | .62 | .60 | .78 | 1.00 |
| **11** | Eat eggs during breastfeeding† | .49 | .43 | .45 | .52 | .62 | .73 | .73 | .68 | .70 | .67 |
| **12** | Consume milk or milk products during breastfeeding† | .44 | .38 | .38 | .49 | .58 | .69 | .75 | .68 | .65 | .67 |
| **13** | Eat meat, poultry, or fish during breastfeeding† | .47 | .44 | .43 | .53 | .63 | .69 | .75 | .75 | .67 | .64 |
| **14** | Whether child gets vaccinations† | .40 | .35 | .30 | .42 | .42 | .43 | .46 | .43 | .45 | .46 |
| **15** | Feed child when sick | .44 | .37 | .34 | .42 | .46 | .48 | .49 | .49 | .48 | .51 |
| **16** | Whether to breastfeed child | .42 | .32 | .30 | .38 | .42 | .38 | .36 | .35 | .48 | .47 |
| **17** | When to stop breastfeeding | .37 | .31 | .27 | .37 | .37 | .34 | .34 | .31 | .41 | .44 |
| **18** | Introducing foods and liquids | .39 | .32 | .24 | .40 | .41 | .35 | .38 | .34 | .41 | .43 |
| **19** | Child is fed special foods for children | .35 | .28 | .22 | .35 | .39 | .43 | .44 | .42 | .40 | .40 |
| **20** | Child is offered eggs | .37 | .32 | .40 | .36 | .40 | .48 | .51 | .47 | .48 | .43 |
| **21** | Child is offered milk or milk products | .42 | .30 | .38 | .40 | .45 | .54 | .57 | .55 | .51 | .49 |
| **22** | Child is offered meat, poultry, or fish | .39 | .30 | .36 | .39 | .44 | .53 | .54 | .54 | .49 | .47 |
| **23** | Go to doctor when ill | .45 | .42 | .34 | .47 | .39 | .38 | .42 | .44 | .36 | .32 |
| **24** | Have another child† | .32 | .34 | .36 | .40 | .35 | .34 | .34 | .38 | .33 | .34 |
| **25** | Use contraceptive methods† | .27 | .36 | .38 | .35 | .33 | .35 | .32 | .34 | .30 | .29 |
| **26** | Go to doctor during pregnancy | .43 | .38 | .32 | .56 | .46 | .45 | .49 | .51 | .47 | .40 |
| **27** | Whether to take sick child to doctor | .34 | .41 | .40 | .41 | .41 | .43 | .47 | .50 | .43 | .41 |
| **28** | Whether to take child for well visits | .40 | .37 | .29 | .41 | .41 | .47 | .48 | .49 | .46 | .47 |
| **29** | Who cares for child when you are outside the home† | .39 | .38 | .23 | .39 | .38 | .36 | .37 | .40 | .40 | .40 |

† Item not used in indicator construction

|  | **11** | **12** | **13** | **14** | **15** | **16** | **17** | **18** | **19** | **20** | **21** | **22** | **23** |
| --- | --- | --- | --- | --- | --- | --- | --- | --- | --- | --- | --- | --- | --- |
| **11** | 1.00 |  |  |  |  |  |  |  |  |  |  |  |  |
| **12** | .82 | 1.00 |  |  |  |  |  |  |  |  |  |  |  |
| **13** | .78 | .80 | 1.00 |  |  |  |  |  |  |  |  |  |  |
| **14** | .44 | .44 | .47 | 1.00 |  |  |  |  |  |  |  |  |  |
| **15** | .46 | .43 | .45 | .63 | 1.00 |  |  |  |  |  |  |  |  |
| **16** | .42 | .40 | .39 | .44 | .49 | 1.00 |  |  |  |  |  |  |  |
| **17** | .39 | .41 | .37 | .42 | .45 | .80 | 1.00 |  |  |  |  |  |  |
| **18** | .41 | .39 | .36 | .36 | .41 | .70 | .70 | 1.00 |  |  |  |  |  |
| **19** | .40 | .44 | .42 | .39 | .37 | .51 | .54 | .58 | 1.00 |  |  |  |  |
| **20** | .54 | .54 | .51 | .45 | .44 | .44 | .40 | .43 | .48 | 1.00 |  |  |  |
| **21** | .58 | .59 | .56 | .50 | .50 | .47 | .44 | .47 | .54 | .85 | 1.00 |  |  |
| **22** | .55 | .56 | .56 | .47 | .48 | .46 | .42 | .46 | .54 | .79 | .88 | 1.00 |  |
| **23** | .35 | .31 | .39 | .38 | .39 | .24 | .23 | .27 | .29 | .32 | .31 | .32 | 1.00 |
| **24** | .34 | .30 | .36 | .36 | .34 | .19 | .20 | .23 | .21 | .29 | .29 | .31 | .54 |
| **25** | .31 | .30 | .32 | .33 | .29 | .21 | .20 | .20 | .24 | .38 | .34 | .35 | .44 |
| **26** | .41 | .36 | .44 | .43 | .41 | .29 | .27 | .30 | .31 | .31 | .34 | .35 | .71 |
| **27** | .47 | .44 | .49 | .52 | .48 | .32 | .29 | .33 | .30 | .49 | .46 | .46 | .59 |
| **28** | .42 | .42 | .45 | .65 | .66 | .40 | .38 | .41 | .44 | .47 | .51 | .49 | .49 |
| **29** | .32 | .31 | .35 | .49 | .53 | .31 | .29 | .33 | .36 | .42 | .44 | .41 | .43 |

|  | **24** | **25** | **26** | **27** | **28** | **29** |
| --- | --- | --- | --- | --- | --- | --- |
| **24** | 1.00 |  |  |  |  |  |
| **25** | .74 | 1.00 |  |  |  |  |
| **26** | .50 | .41 | 1.00 |  |  |  |
| **27** | .47 | .43 | .58 | 1.00 |  |  |
| **28** | .41 | .39 | .51 | .61 | 1.00 |  |
| **29** | .32 | .26 | .45 | .49 | .58 | 1.00 |

**Appendix Table A6.** Pairwise correlations between survey items in EFA on health products

*Source: Respondents of women from dual-adult households, from a random half sample of data from the SELEVER and TRAIN projects*

|  |  | **Survey item** | **1** | **2** | **3** | **4** | **5** | **6** | **7** | **8** |
| --- | --- | --- | --- | --- | --- | --- | --- | --- | --- | --- |
| Respondent contributes to the decision to purchase | **1** | Small foods | 1.00 |  |  |  |  |  |  |  |
|  | **2** | Large foods | .79 | 1.00 |  |  |  |  |  |  |
|  | **3** | Eggs | .70 | .73 | 1.00 |  |  |  |  |  |
|  | **4** | Milk | .68 | .71 | .83 | 1.00 |  |  |  |  |
|  | **5** | Meat/poultry/fish | .69 | .74 | .78 | .81 | 1.00 |  |  |  |
|  | **6** | Special foods for children† | .51 | .49 | .51 | .53 | .55 | 1.00 |  |  |
|  | **7** | Nutritious program foods† | .47 | .48 | .48 | .51 | .49 | .60 | 1.00 |  |
|  | **8** | Medicine for child | .54 | .55 | .56 | .58 | .59 | .74 | .57 | 1.00 |
|  | **9** | Medicine for self | .52 | .53 | .53 | .55 | .55 | .49 | .50 | .58 |
|  | **10** | Clothing for child† | .50 | .49 | .47 | .51 | .50 | .66 | .48 | .74 |
|  | **11** | Clothing for self† | .49 | .48 | .49 | .52 | .50 | .46 | .48 | .53 |
|  | **12** | Toiletries | .56 | .52 | .57 | .59 | .57 | .48 | .46 | .51 |
| Respondent is typically able to acquire the product by some means | **13** | Small foods | .12 | .09 | .10 | .09 | .09 | .08 | .05 | .09 |
|  | **14** | Large foods | .08 | .15 | .11 | .10 | .12 | .07 | .08 | .11 |
|  | **15** | Eggs | .12 | .10 | .14 | .10 | .12 | .10 | .05 | .12 |
|  | **16** | Milk | .12 | .10 | .11 | .11 | .11 | .09 | .04 | .10 |
|  | **17** | Meat/poultry/fish | .11 | .11 | .12 | .09 | .14 | .08 | .05 | .10 |
|  | **18** | Special foods for children† | .14 | .14 | .12 | .09 | .13 | .15 | .10 | .17 |
|  | **19** | Nutritious program foods† | .10 | .11 | .09 | .06 | .09 | .09 | .12 | .10 |
|  | **20** | Medicine for child | .08 | .08 | .07 | .03 | .08 | .05 | .01 | .10 |
|  | **21** | Medicine for self | .05 | .07 | .04 | .04 | .06 | .03 | .02 | .07 |
|  | **22** | Clothing for child† | .01 | .00 | .01 | .02 | -.01 | .01 | .00 | .03 |
|  | **23** | Clothing for self† | .02 | .01 | .00 | -.01 | .00 | .00 | .01 | .02 |
|  | **24** | Toiletries | .07 | .05 | .05 | .05 | .06 | .02 | .00 | .04 |

† Item not used in indicator construction

|  | **9** | **10** | **11** | **12** | **13** | **14** | **15** | **16** | **17** | **18** | **19** | **20** | **21** |
| --- | --- | --- | --- | --- | --- | --- | --- | --- | --- | --- | --- | --- | --- |
| **9** | 1.00 |  |  |  |  |  |  |  |  |  |  |  |  |
| **10** | .57 | 1.00 |  |  |  |  |  |  |  |  |  |  |  |
| **11** | .66 | .67 | 1.00 |  |  |  |  |  |  |  |  |  |  |
| **12** | .59 | .58 | .69 | 1.00 |  |  |  |  |  |  |  |  |  |
| **13** | .12 | .14 | .14 | .12 | 1.00 |  |  |  |  |  |  |  |  |
| **14** | .11 | .12 | .14 | .11 | .63 | 1.00 |  |  |  |  |  |  |  |
| **15** | .12 | .14 | .14 | .11 | .57 | .52 | 1.00 |  |  |  |  |  |  |
| **16** | .13 | .13 | .13 | .10 | .58 | .51 | .81 | 1.00 |  |  |  |  |  |
| **17** | .11 | .11 | .13 | .10 | .62 | .61 | .66 | .68 | 1.00 |  |  |  |  |
| **18** | .10 | .16 | .15 | .11 | .50 | .51 | .61 | .63 | .66 | 1.00 |  |  |  |
| **19** | .09 | .10 | .13 | .07 | .53 | .56 | .59 | .60 | .65 | .77 | 1.00 |  |  |
| **20** | .12 | .12 | .12 | .07 | .56 | .50 | .59 | .62 | .65 | .67 | .71 | 1.00 |  |
| **21** | .12 | .12 | .13 | .08 | .64 | .65 | .61 | .63 | .72 | .71 | .75 | .84 | 1.00 |
| **22** | .07 | .10 | .10 | .06 | .57 | .51 | .45 | .46 | .56 | .53 | .55 | .63 | .76 |
| **23** | .07 | .08 | .11 | .06 | .56 | .53 | .44 | .44 | .58 | .50 | .53 | .58 | .72 |
| **24** | .10 | .09 | .11 | .12 | .60 | .47 | .57 | .60 | .62 | .56 | .58 | .67 | .75 |
|  |  |  |  |  |  |  |  |  |  |  |  |  |  |
|  | **22** | **23** | **24** |  |  |  |  |  |  |  |  |  |  |
| **22** | 1.00 |  |  |  |  |  |  |  |  |  |  |  |  |
| **23** | .83 | 1.00 |  |  |  |  |  |  |  |  |  |  |  |
| **24** | .64 | .65 | 1.00 |  |  |  |  |  |  |  |  |  |  |

**Appendix Table A7.** Factor loadings from EFA of women’s input into women and child health and nutrition items TRAIN (Bangladesh) and SELEVER (Burkina Faso)

|  | **TRAIN, Bangladesh** | | | | | **SELEVER, Burkina Faso** | | | | | | | |  |
| --- | --- | --- | --- | --- | --- | --- | --- | --- | --- | --- | --- | --- | --- | --- |
| **Survey item** | Factor 1: Decides on own health and diet, including during pregnancy and lactation | Factor 2: Decides on child's health and diet | | Factor 3: Decides to seek healthcare | | | Factor 1: Decides on own health and diet, including during pregnancy and lactation | Factor 2: Decides on child's health and diet | | | Factor 3: Decides to seek healthcare | | Factor 4: Decides on feeding child animal source foods | |
| Rest when ill | .51 | | * | | * | .43 | | | * | * | | * | |  |
| Foods to prepare | .52 | | * | | * | * | | | * | * | | * | |  |
| Foods to eat^1^ | .52 | | * | | * | * | | | * | * | | * | |  |
| Work during pregnancy | .49 | | * | | * | .77 | | | * | * | | * | |  |
| Rest during pregnancy | .78 | | * | | * | .77 | | | * | * | | * | |  |
| Eggs during pregnancy | .82 | | * | | * | .85 | | | * | * | | * | |  |
| Milk or milk products during pregnancy | .90 | | * | | * | .78 | | | * | * | | * | |  |
| Meat, poultry, or fish during pregnancy | .73 | | * | | * | .86 | | | * | * | | * | |  |
| Work during breastfeeding | .75 | | * | | * | .82 | | | * | * | | * | |  |
| Rest during breastfeeding | .64 | | * | | * | .80 | | | * | * | | * | |  |
| Eggs during breastfeeding | .82 | | * | | * | .81 | | | * | * | | * | |  |
| Milk or milk products during breastfeeding | .79 | | * | | * | .83 | | | * | * | | * | |  |
| Meat, poultry, or fish during breastfeeding | .83 | | * | | * | .80 | | | * | * | |  | |  |
| Whether child vaccinated | * | | .42 | | * | * | | | * | * | | * | |  |
| Feed child when sick | * | | .44 | | * | * | | | .41 | * | | * | |  |
| Whether to breastfeed | * | | .75 | | * | * | | | .94 | * | | * | |  |
| When to stop breastfeeding | * | | .76 | | * | * | | | .90 | * | | * | |  |
| Introducing foods and liquids | * | | .73 | | * | * | | | .93 | * | | * | |  |
| Child is fed special foods for children | * | | .58 | | * | * | | | .52 | * | |  | |  |
| Child is offered eggs | * | | .73 | | * | * | | | * | * | | .74 | |  |
| Child is offered milk or milk products | * | | .72 | | * | * | | | * | * | | .81 | |  |
| Child is offered meat, poultry, or fish | * | | .68 | | * | * | | | * | * | | .75 | |  |
| Go to doctor when ill | * | | * | | .72 | * | | | * | .77 | | * | |  |
| Have another child | * | | * | | .80 | * | | | * | .66 | | * | |  |
| Use contraceptives | * | | * | | .77 | * | | | * | .48 | | * | |  |
| Foods you can eat^1^ | * | | * | | * | * | | | * | .56 | | * | |  |
| Go to doctor during pregnancy | * | | * | | .70 | * | | | * | .57 | | * | |  |
| Whether to take sick child to doctor | * | | * | | .47 | * | | | * | .72 | | * | |  |
| Whether to take child for well visits | * | | .43 | | .52 | * | | | * | .43 | | * | |  |
| Who cares for child when you are outside the home | * | | * | | .45 | * | | | * | * | | * | |  |

**Note:** Sample is a randomly selected half and includes only women from dual-adult households who have been pregnant or given birth in the last two years and have a child under age 2. EFA models were based on variance-covariance matrices and missing data was imputed using the expectation-maximization algorithm. Factor loadings are from an oblique (oblimin) rotation.

*Factor loadings < 0.4.

-- Item omitted by project.

^1^ Item loaded on different factors for SELEVER and TRAIN.

**Appendix Table A8.** Factor loadings from EFA of health products for TRAIN (Bangladesh) and SELEVER (Burkina Faso)

|  | **TRAIN** | | **SELEVER** | |
| --- | --- | --- | --- | --- |
|  | **Bangladesh** | | **Burkina Faso** | |
| Survey item | Factor 1: Decides to purchase food and health products | Factor 2: Has accesses food and health products | Factor 1: Decides to purchase food and health products | Factor 2: Has accesses food and health products |
| Respondent contributes to the decision to purchase: |  |  |  |  |
| Small foods | .89 | * | .83 | * |
| Large foods | .91 | * | .88 | * |
| Eggs | .91 | * | .94 | * |
| Milk | .94 | * | .93 | * |
| Meat/poultry/fish | .94 | * | .94 | * |
| Special foods for children | .91 | * | .95 | * |
| Nutritious program foods | .90 | * | .94 | * |
| Medicine for child | .92 | * | .95 | * |
| Medicine for self | .90 | * | .91 | * |
| Clothing for child | .88 | * | -- | -- |
| Clothing for self | .85 | * | .88 | * |
| Toiletries | .86 | * | .90 | * |
| Respondent is typically able to acquire the product by some means: |  |  |  | * |
| Small foods | * | .94 | * | .77 |
| Large foods | * | .93 | * | .60 |
| Eggs | * | .93 | * | .85 |
| Milk | * | .93 | * | .88 |
| Meat/poultry/fish | * | .97 | * | .91 |
| Special foods for children | * | .96 | * | .92 |
| Nutritious program foods | * | .97 | * | .92 |
| Medicine for child | * | .98 | * | .91 |
| Medicine for self | * | .98 | * | .87 |
| Clothing for child | * | .95 | -- | -- |
| Clothing for self | * | .94 | * | .85 |
| Toiletries | * | .97 | * | .87 |

**Note:** Sample is a randomly selected half and includes only women from dual-adult households. Factor loadings are from an orthogonal (varimax) rotation. EFA models were based on tetrachoric correlation matrices.

*Factor loading < 0.4.

-- Item omitted by project.

**Appendix Table A9.** Differentiation between Purchasing vs Access indicators

|  | Decides to purchase and has access to | Only decides to purchase | Only has access to | Cannot decide to purchase and does not have access to |
| --- | --- | --- | --- | --- |
|  | Mean | Mean | Mean | Mean |
|  | (SE) | (SE) | (SE) | (SE) |
| Small foods | 0.61 | 0.05 | 0.29 | 0.05 |
|  | (0.01) | (0.00) | (0.01) | (0.00) |
| Large foods | 0.47 | 0.10 | 0.30 | 0.13 |
|  | (0.01) | (0.01) | (0.01) | (0.01) |
| Eggs | 0.65 | 0.05 | 0.26 | 0.05 |
|  | (0.01) | (0.00) | (0.01) | (0.00) |
| Milk | 0.69 | 0.06 | 0.22 | 0.03 |
|  | (0.01) | (0.00) | (0.01) | (0.00) |
| Meat/poultry/fish | 0.58 | 0.08 | 0.27 | 0.07 |
|  | (0.01) | (0.00) | (0.01) | (0.00) |
| Medicine for children | 0.63 | 0.09 | 0.22 | 0.06 |
|  | (0.01) | (0.00) | (0.01) | (0.00) |
| Medicine for self | 0.75 | 0.08 | 0.12 | 0.05 |
|  | (0.01) | (0.00) | (0.01) | (0.00) |
| Toiletries | 0.75 | 0.05 | 0.17 | 0.03 |
|  | (0.01) | (0.00) | (0.00) | (0.00) |

**Appendix Table A10.** Pairwise correlations between survey items in CFA on women and children health and nutrition decisions

*Source: Responses of women from dual-adult households, from a random half sample of data from the SELEVER and TRAIN projects and all data from the ANGeL, FAARM, Grameen, and WorldVeg projects*

|  | **Survey item** | | | **1** | | **2** | | **3** | | **4** | | **5** | | **6** | | **7** | | **8** | | **9** | | **10** |
| --- | --- | --- | --- | --- | --- | --- | --- | --- | --- | --- | --- | --- | --- | --- | --- | --- | --- | --- | --- | --- | --- | --- |
| **1** | Rest when ill | | | 1.00 | |  | |  | |  | |  | |  | |  | |  | |  | |  |
| **2** | Foods to prepare | | | .43 | | 1.00 | |  | |  | |  | |  | |  | |  | |  | |  |
| **3** | Foods you can eat | | | .39 | | .79 | | 1.00 | |  | |  | |  | |  | |  | |  | |  |
| **4** | Work during pregnancy | | | .60 | | .47 | | .42 | | 1.00 | |  | |  | |  | |  | |  | |  |
| **5** | Rest during pregnancy | | | .60 | | .47 | | .43 | | .81 | | 1.00 | |  | |  | |  | |  | |  |
| **6** | Eat eggs during pregnancy | | | .53 | | .43 | | .45 | | .67 | | .69 | | 1.00 | |  | |  | |  | |  |
| **7** | Consume milk or milk products during pregnancy | | | .53 | | .43 | | .44 | | .67 | | .69 | | .85 | | 1.00 | |  | |  | |  |
| **8** | Eat meat, poultry, or fish during pregnancy | | | .53 | | .43 | | .43 | | .64 | | .65 | | .81 | | .84 | | 1.00 | |  | |  |
| **9** | Child is offered eggs | | | .43 | | .32 | | .33 | | .44 | | .48 | | .54 | | .57 | | .51 | | 1.00 | |  |
| **10** | Child is offered milk or milk products | | | .44 | | .32 | | .32 | | .46 | | .50 | | .54 | | .56 | | .53 | | .88 | | 1.00 |
| **11** | Child is offered meat, poultry, or fish | | | .44 | | .31 | | .32 | | .46 | | .49 | | .53 | | .57 | | .55 | | .82 | | .88 |
| **12** | Feed child when sick | | | .48 | | .41 | | .38 | | .53 | | .53 | | .51 | | .52 | | .51 | | .59 | | .61 |
| **13** | Whether to breastfeed child | | | .32 | | .28 | | .29 | | .32 | | .33 | | .32 | | .32 | | .29 | | .61 | | .64 |
| **14** | When to stop breastfeeding | | | .25 | | .27 | | .28 | | .33 | | .35 | | .33 | | .34 | | .30 | | .34 | | .34 |
| **15** | Introducing food and liquids | | | .32 | | .34 | | .31 | | .43 | | .47 | | .46 | | .48 | | .42 | | .48 | | .50 |
| **16** | Go to doctor when ill | | | .54 | | .49 | | .44 | | .47 | | .42 | | .42 | | .44 | | .48 | | .33 | | .33 |
| **17** | Have another child† | | | .51 | | .27 | | .26 | | .36 | | .35 | | .40 | | .40 | | .43 | | .39 | | .39 |
| **18** | Use contraceptive methods† | | | .45 | | .24 | | .22 | | .34 | | .34 | | .36 | | .38 | | .40 | | .40 | | .42 |
| **19** | Go to doctor during pregnancy | | | .50 | | .47 | | .39 | | .60 | | .56 | | .49 | | .53 | | .55 | | .40 | | .43 |
| **20** | Whether to take sick child to doctor | | | .47 | | .51 | | .49 | | .50 | | .49 | | .49 | | .52 | | .51 | | .46 | | .46 |
| **21** | Whether to take child for well visits | | | .48 | | .47 | | .45 | | .50 | | .49 | | .47 | | .51 | | .52 | | .49 | | .49 |
|  | **11** | **12** | **13** | | **14** | | **15** | | **16** | | **17** | | **18** | | **19** | | **20** | | **21** | |  |  |
| **11** | 1.00 |  |  | |  | |  | |  | |  | |  | |  | |  | |  | |  |  |
| **12** | .60 | 1.00 |  | |  | |  | |  | |  | |  | |  | |  | |  | |  |  |
| **13** | .64 | .55 | 1.00 | |  | |  | |  | |  | |  | |  | |  | |  | |  |  |
| **14** | .35 | .40 | .73 | | 1.00 | |  | |  | |  | |  | |  | |  | |  | |  |  |
| **15** | .50 | .49 | .56 | | .66 | | 1.00 | |  | |  | |  | |  | |  | |  | |  |  |
| **16** | .34 | .41 | .28 | | .22 | | .29 | | 1.00 | |  | |  | |  | |  | |  | |  |  |
| **17** | .39 | .38 | .25 | | .20 | | .23 | | .48 | | 1.00 | |  | |  | |  | |  | |  |  |
| **18** | .41 | .34 | .23 | | .23 | | .24 | | .41 | | .76 | | 1.00 | |  | |  | |  | |  |  |
| **19** | .43 | .46 | .29 | | .26 | | .34 | | .62 | | .48 | | .41 | | 1.00 | |  | |  | |  |  |
| **20** | .47 | .55 | .37 | | .28 | | .37 | | .65 | | .44 | | .39 | | .60 | | 1.00 | |  | |  |  |
| **21** | .51 | .65 | .44 | | .33 | | .41 | | .55 | | .42 | | .38 | | .57 | | .71 | | 1.00 | |  |  |

**Appendix Table A11.** Pairwise correlations between survey items in CFA on health products

|  |  | **Survey item** | **1** | **2** | **3** | **4** | **5** | **6** | **7** | **8** | **9** | **10** |
| --- | --- | --- | --- | --- | --- | --- | --- | --- | --- | --- | --- | --- |
| Respondent contributes to the decision to purchase | **1** | Small foods | 1.00 |  |  |  |  |  |  |  |  |  |
|  | **2** | Large foods | .77 | 1.00 |  |  |  |  |  |  |  |  |
|  | **3** | Eggs | .70 | .71 | 1.00 |  |  |  |  |  |  |  |
|  | **4** | Milk | .70 | .71 | .83 | 1.00 |  |  |  |  |  |  |
|  | **5** | Meat/poultry/fish | .71 | .75 | .80 | .79 | 1.00 |  |  |  |  |  |
|  | **6** | Medicine for child | .50 | .52 | .53 | .52 | .57 | 1.00 |  |  |  |  |
|  | **7** | Medicine for self | .49 | .50 | .49 | .51 | .52 | .57 | 1.00 |  |  |  |
|  | **8** | Toiletries | .54 | .53 | .55 | .58 | .55 | .47 | .55 | 1.00 |  |  |
| Respondent is typically able to acquire the product by some means | **9** | Small foods | .15 | .09 | .09 | .08 | .10 | .10 | .14 | .13 | 1.00 |  |
|  | **10** | Large foods | .13 | .17 | .14 | .11 | .14 | .15 | .14 | .13 | .65 | 1.00 |
|  | **11** | Eggs | .14 | .12 | .17 | .14 | .13 | .13 | .12 | .13 | .60 | .58 |
|  | **12** | Milk | .15 | .12 | .14 | .14 | .12 | .11 | .13 | .13 | .61 | .57 |
|  | **13** | Meat/poultry/fish | .10 | .10 | .11 | .08 | .14 | .09 | .09 | .09 | .62 | .62 |
|  | **14** | Medicine for child | .08 | .06 | .06 | .03 | .06 | .10 | .14 | .07 | .59 | .55 |
|  | **15** | Medicine for self | .10 | .08 | .08 | .08 | .09 | .12 | .13 | .13 | .66 | .68 |
|  | **16** | Toiletries | .08 | .04 | .06 | .06 | .04 | .05 | .10 | .12 | .62 | .50 |

*Source: Respondents of women from dual-adult households, from a random half sample of data from the SELEVER and TRAIN projects and all data from the ANGeL, FAARM, Grameen, and WorldVeg projects*

† Item not used in indicator construction

|  | **11** | **12** | **13** | **14** | **15** | **16** |
| --- | --- | --- | --- | --- | --- | --- |
| **11** | 1.00 |  |  |  |  |  |
| **12** | .80 | 1.00 |  |  |  |  |
| **13** | .67 | .69 | 1.00 |  |  |  |
| **14** | .56 | .61 | .64 | 1.00 |  |  |
| **15** | .64 | .68 | .72 | .87 | 1.00 |  |
| **16** | .57 | .60 | .62 | .71 | .78 | 1.00 |

**Appendix Table A12**. Tests of measurement invariance between mothers and non-mothers for health products factors

| **Test statistic** | **TRAIN** | **ANGeL** | **FAARM** | **SELEVER** | **WorldVeg** |
| --- | --- | --- | --- | --- | --- |
| Overall |  |  |  |  |  |
| CFI | .984 | .987 | .883 | .944 | .978 |
| TLI | .978 | .978 | .833 | .926 | .969 |
| RMSEA | .051 | .049 | .117 | .075 | .063 |
| Configural invariance |  |  |  |  |  |
| CFI | .981 | .987 | .890 | .940 | .976 |
| RMSEA | .054 | .049 | .114 | .085 | .066 |
| Weak invariance |  |  |  |  |  |
| CFI | .981 | .987 | .887 | .941 | .974 |
| RMSEA | .053 | .047 | .111 | .081 | .066 |
| Strong invariance |  |  |  |  |  |
| CFI | .980 | .987 | .889 | .942 | .974 |
| RMSEA | .052 | .046 | .106 | .076 | .063 |
| Strict invariance |  |  |  |  |  |
| CFI | .977 | .984 | .868 | .941 | .968 |
| RMSEA | .053 | .048 | .110 | .073 | .068 |
| Mean invariance |  |  |  |  |  |
| CFI | .976 | .984 | .869 | .940 | .968 |
| RMSEA | .054 | .048 | .109 | .073 | .068 |

Note: Multigroup CFA models include respondents from dual-adult households only and were run using standardized estimates and full information maximum likelihood estimation. Models using data from the Grameen project did not converge.

**Appendix Table 13.** Redundancy among health and nutrition indicators

|  | Decides on own health and diet | Decides on health and diet during pregnancy | Decides on child’s diet | Decides on weaning and breastfeeding | Decides to seek healthcare | Decides to purchase food and health products | Has access to food and health products |
| --- | --- | --- | --- | --- | --- | --- | --- |
| Decides on own health and diet | 1.00 |  |  |  |  |  |  |
| Decides on health and diet during pregnancy | .57 | 1.00 |  |  |  |  |  |
| Decides on child's diet | .42 | .56 | 1.00 |  |  |  |  |
| Decides on weaning and breastfeeding | .42 | .61 | .51 | 1.00 |  |  |  |
| Decides to seek healthcare | .82 | .79 | .66 | .66 | 1.00 |  |  |
| Decides to purchase food and health products | .73 | .68 | .55 | .61 | .64 | 1.00 |  |
| Has access to food and health products | .39 | .41 | .31 | .34 | .48 | .65 | 1.00 |

**Appendix Table A14.** Associations (Cramer’s V) between core pro-WEAI indicators and health and nutrition indicators

|  |  | Autonomy | Self-efficacy | Never violence | Produc-tive  deci-  sions | Asset owner-ship | Finan-  cial  serv-  ices | Control over income | Work balance | Mobility | Group member-ship | Group influence | Respect |
| --- | --- | --- | --- | --- | --- | --- | --- | --- | --- | --- | --- | --- | --- |
| **All projects** | Decides on own health and diet | .07 | .08 | .05 | .23 | .04 | .15 | .06 | .04 | .02 | .03 | .03 | .01 |
|  | Decides on health and diet during pregnancy | .05 | .12 | .01 | .19 | .04 | .11 | .07 | .03 | .01 | .07 | .07 | .00 |
|  | Decides on child’s diet | .03 | .04 | .00 | .10 | .02 | .02 | .03 | .10 | .03 | .02 | .01 | .02 |
|  | Decides on weaning and breastfeeding | .04 | .05 | .03 | .11 | .00 | .11 | .08 | .03 | .01 | .08 | .08 | .01 |
|  | Decides to seek healthcare | .06 | .10 | .04 | .15 | .07 | .00 | .10 | .06 | .01 | .04 | .04 | .07 |
|  | Decides to purchase food and health products | .07 | .11 | .01 | .17 | .06 | .29 | .16 | .09 | .02 | .02 | .03 | .05 |
|  | Access to food and health products | .03 | .08 | .07 | .13 | .06 | .12 | .07 | .09 | .02 | .03 | .01 | .03 |
|  |  |  |  |  |  |  |  |  |  |  |  |  |  |
| **Bangladesh** | Decides on own health and diet | .05 | .09 | .00 | .27 | .12 | .13 | .13 | .04 | .03 | .08 | .06 | .02 |
|  | Decides on health and diet during pregnancy | .03 | .12 | .00 | .19 | .06 | .05 | .10 | .01 | .03 | .01 | .00 | .01 |
|  | Decides on child’s diet | .00 | .06 | .02 | .11 | .03 | .05 | .06 | .12 | .00 | .03 | .03 | .00 |
|  | Decides on weaning and breastfeeding | .01 | .06 | .00 | .13 | .03 | .05 | .11 | .01 | .04 | .00 | .02 | .00 |
|  | Decides to seek healthcare | .07 | .14 | .02 | .15 | .07 | .13 | .20 | .09 | .02 | .01 | .02 | .06 |
|  | Decides to purchase food and health products | .10 | .10 | .02 | .19 | .11 | .22 | .16 | .10 | .07 | .10 | .04 | .04 |
|  | Access to food and health products | .01 | .04 | .07 | .15 | .08 | .12 | .08 | .11 | .02 | .01 | .03 | .01 |
|  |  |  |  |  |  |  |  |  |  |  |  |  |  |
| **Burkina Faso and Mali** | Decides on own health and diet | .01 | .05 | .01 | .10 | .01 | .11 | .11 | .03 | .03 | .11 | .08 | .08 |
|  | Decides on health and diet during pregnancy | .07 | .11 | .24 | .15 | .07 | .18 | .08 | .06 | .07 | .12 | .12 | .00 |
|  | Decides on child’s diet | .06 | .02 | .02 | .04 | .06 | .02 | .03 | .08 | .05 | .10 | .10 | .02 |
|  | Decides on weaning and breastfeeding | .03 | .02 | .17 | .07 | .07 | .13 | .03 | .07 | .04 | .10 | .10 | .01 |
|  | Decides to seek healthcare | .08 | .01 | .09 | .16 | .04 | .06 | .03 | .02 | .05 | .08 | .07 | .03 |
|  | Decides to purchase food and health products | .11 | .12 | .20 | .07 | .10 | .23 | .09 | .00 | .06 | .08 | .08 | .03 |
|  | Access to food and health products | .03 | .15 | .01 | .01 | .06 | .07 | .05 | .04 | .05 | .02 | .01 | .06 |

**Appendix Figure A1.** Percent of respondents adequate in each indicator using alternative indicator cutoffs

**Source:** Project data from ANGeL (N=3,917), FAARM (N=287), Grameen (N=380), SELEVER (N=1,777), TRAIN (N=5,040), and WorldVeg (N=714)

**Note:** Weighted by the inverse of project sample size, so that characteristics are equally weighted by project sample. “Participates” means the respondent participates solely or jointly in decisions related to that indicator; “medium” means that respondent is the sole decision-maker or feels she can contribute to the decision to at least a medium extent; “high” means that the respondent is the sole decision-maker or feels she can contribute to the decision to a high extent. The percent of products indicates the percent of eight food and health products that the respondent has the freedom to purchase or ability to access. The textured orange bars indicate the selected cutoff for each indicator. Each of the seven indicators presented is comprised of at least three items.

**Appendix Figure A2.** Percent of women achieving adequacy, by region, indicator, and age group

* p<.05; ** p<.01

***Note:*** Analyses use project data from Bangladesh: ANGeL (N=3,917), FAARM (N=287), and TRAIN (N=5,040); and Burkina Faso and Mali: Grameen (N=380), SELEVER (1,777), and WorldVeg (N=714) and are weighted by inverse project sample size. The two older age groups were collapsed for Bangladesh, because very few women were in the oldest age group. For the Bangladesh samples, post-ANOVA contrasts reveal that for ‘decides on own health and diet’, ‘decides on child’s diet’, ‘decides to seek healthcare’, ‘decides to purchase food and health products’, and ‘has access to food and health products’ all age groups are significantly different from one another; for ‘decides on health and diet during pregnancy’ and ‘decides on weaning and breastfeeding’ 25-35 years differs significantly from 16-24 years. For the Burkina Faso and Mali sample, post-ANOVA contrasts reveal that for ‘decides on child’s diet’ and ‘decides to seek healthcare’ 35-44 years is significantly different from 25-34 years and from 45+ years; for ‘decides on weaning and breastfeeding’ 45+ years differs significantly from 35-44 years; and for ‘decides to purchase food and health products’ 35-44 years differs significantly from 25-34 years. Each of the seven indicators presented is comprised of at least three items.

**Appendix Figure A3.** Percent of women achieving adequacy, by region, indicator, and highest level of education achieved

* p<.05; ** p<.01

***Note:*** Analysis use project data from Bangladesh: ANGeL (N=3,917), FAARM (N=287), and TRAIN (N=5,040); and Burkina Faso/Mali: Grameen (N=380), SELEVER (1,777), and WorldVeg (N=714) and are weighted by inverse project sample size. The primary and secondary education categories were collapsed in Burkina Faso and Mali, because very few women had completed secondary education. For the Bangladesh samples, post-ANOVA contrasts reveal that for ‘decides on health and diet during pregnancy’, ‘decides on child’s diet’, ‘decides to seek healthcare’ and ‘has access to food and health products’ the primary education group differs significantly from the less than primary education group; for ‘decides to purchase food and health products’ the less than primary education group differs significantly from the never attended school group. For the Burkina Faso and Mali sample, post-ANOVA contrasts reveal that for ‘decides on child’s diet and ‘decides to purchase food and health products’ the less than primary education group differs significantly from the never attended school group. Each of the seven indicators presented is comprised of at least three items.
